# Development of a Hypoxia-Triggered Supramolecular Nanoplatform for Synergistic Hypoxia Alleviation and Amplified Photodynamic Cancer Therapy

**DOI:** 10.3390/molecules31142433

**Published:** 2026-07-11

**Authors:** Ningning Luo, Yiliang Wu, Jiaxin Zheng, Chi Zhang, Xiaoyang Qian, Caoqing Ji, Aiqing Jiang, Yong Ling, Xin Liu

**Affiliations:** 1 Jiangsu Province Key Laboratory for Inflammation and Molecular Drug Target, School of Pharmacy, Nantong University, Nantong 226001, China; 2 Jiangsu Key Laboratory of Molecular Medicine, Medical School of Nanjing University, Nanjing 210093, China

**Keywords:** photodynamic therapy (PDT), supramolecular assembly, hypoxia-inducible factor 1α (HIF-1α), tumor microenvironment

## Abstract

Photodynamic therapy (PDT) represents a highly promising modality for cancer treatment; however, its clinical success is significantly restricted by the hypoxic nature of the tumor microenvironment (TME). Hypoxia also upregulates hypoxia-inducible factor-1α (HIF-1α) expression, thereby exacerbating tumor malignancy. To tackle this challenge, we engineered a hypoxia-activatable supramolecular nanoplatform (GH@CyNPs) capable of dual hypoxia reversal and amplification of PDT efficacy. The nanoplatform was constructed via host–guest interactions between a water-soluble pillar[5]arene (**WP5**) and an azobenzene-linked cyanine/YC-1 conjugate (**Cy-G**), followed by the co-encapsulation of glucose oxidase (GOx) and catalase (CAT). Upon entering the cancer cells via endocytosis, the azobenzene linker is specifically cleaved by the hypoxic TME, facilitating payload release. Concurrently, the released GOx/CAT pair drives in situ cascade reactions to generate oxygen (O_2_), while YC-1 effectively suppresses HIF-1α expression, thereby achieving synergistic alleviation of hypoxia. Under irradiation, the released cyanine acts as a potent photosensitizer, generating abundant reactive oxygen species (ROS) to kill cancer cells. Both in vitro and in vivo evaluations corroborated that GH@CyNPs exhibit preferential tumor accumulation, profound antitumor efficacy, and excellent biocompatibility. This study presents an innovative paradigm for overcoming TME hypoxia to optimize photodynamic oncotherapy.

## 1. Introduction

Conventional treatment modalities against malignancy, including surgical resection, chemotherapy, and radiotherapy, are frequently compromised by off-target toxicity, severe side effects, and acquired drug resistance [1,2,3]. As an alternative, photodynamic therapy (PDT) has emerged as an attractive, minimally invasive strategy [4,5]. Driven by specific light irradiation, photosensitizers cooperate with local oxygen to generate highly cytotoxic reactive oxygen species (ROS), leading to cell apoptosis and disrupting tumor vasculature with superior spatiotemporal precision and high biosafety [6,7,8]. However, the prevalent hypoxic state of solid tumors remains a formidable obstacle for PDT [9]. Driven by dysfunctional angiogenesis and rapid cancer cell proliferation, over 60% of solid tumors exhibit chronic hypoxia [10,11]. This oxygen-deprived microenvironment severely impairs ROS yield and attenuates the therapeutic efficacy. Extensive research focused on PDT against hypoxic tumors by oxygen delivery [12] or catalytic oxygen production [13]. Nevertheless, these strategies often suffer from premature leakage [14], inadequate catalytic stability [15], or insufficient site-specific drug release [16], failing to sustain continuous oxygen delivery.

Serving as the primary mediator of cellular adaptation to oxygen starvation, hypoxia-inducible factor-1α (HIF-1α), a transcription factor, is pervasively upregulated across a broad spectrum of solid malignancies [17,18]. The pathological hyperactivation of HIF-1α orchestrates the expression of numerous downstream effectors responsible for tumor neovascularization, tissue infiltration, sustained proliferation, and therapeutic resistance [19,20,21]. Consequently, this HIF-1α-driven signaling cascade serves as a key driver of tumor progression and systemic metastasis. Considering the pivotal role of HIF-1α, inhibiting HIF-1α expression is a highly valuable approach to eradicate hypoxia-induced resistance [22]. Notably, lificiguat (YC-1), a well-established and potent HIF-1α inhibitor, robustly blocks HIF-1α accumulation and activation under hypoxic conditions [23,24]. By disrupting these critical oncogenic networks, YC-1 effectively suppresses tumor progression and achieves robust therapeutic efficacy [25,26].

In the realm of supramolecular chemistry, pillar[n]arenes have garnered significant interest as building blocks for smart drug delivery systems, owing to their powerful ability of host–guest recognition, sensitive stimuli-responsiveness, and excellent biological compatibility [27,28,29].

**Scheme 1:**
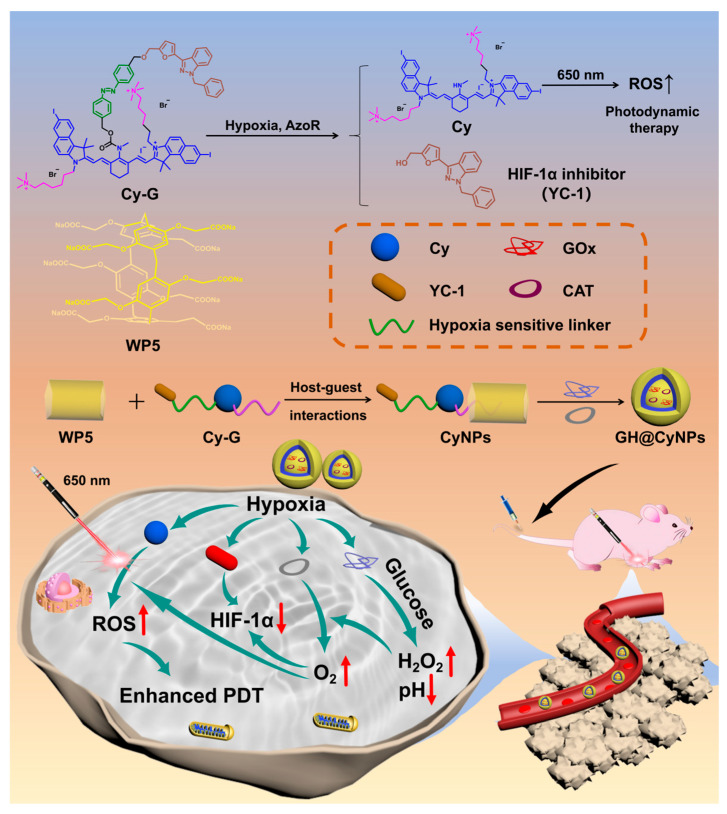
Schematic illustration of GH@CyNPs for dual alleviation of tumor hypoxia and enhanced photodynamic therapy.

Accordingly, we developed a hypoxia-responsive supramolecular nanoplatform (GH@CyNPs) that dual-reverses hypoxia and boosts PDT efficiency. GH@CyNPs self- assembled from water-soluble pillar[5]arene (**WP5**) and an azobenzene-bridged cyanine/YC-1 conjugate (**Cy-G**), further encapsulating GOx and CAT to form nanoparticles (Scheme 1). By virtue of the enhanced permeability and retention (EPR) effect, these nanoparticles accumulate selectively within tumors. The hypoxic tumor microenvironment (TME) induces breakage of the azobenzene linker, resulting in localized cargo release. The cascade of enzymatic oxygen generation (via GOx/CAT) combined with HIF-1α blockade (by YC-1) effectively alleviates tumor hypoxia, thereby maximizing ROS production upon laser irradiation. This intelligent supramolecular nanoplatform exhibits potent tumor treatment effects with a favorable safety profile, offering a new strategy for treating hypoxic tumors.

## 2. Results and Discussion

### 2.1. Preparation and Characterization of GH@CyNPs

The host compound (**WP5**) was synthesized according to the established method [30]. The guest molecule (**Cy-G**) was designed by integrating a quaternary ammonium (as binding sites with **WP5**)-modified cyanine (as the photosensitizer), an azobenzene linker (as the hypoxia-cleavable trigger), and YC-1 (as the HIF-1α inhibitor). The detailed synthetic procedure is shown in the Supporting Information.

**Figure 1:**
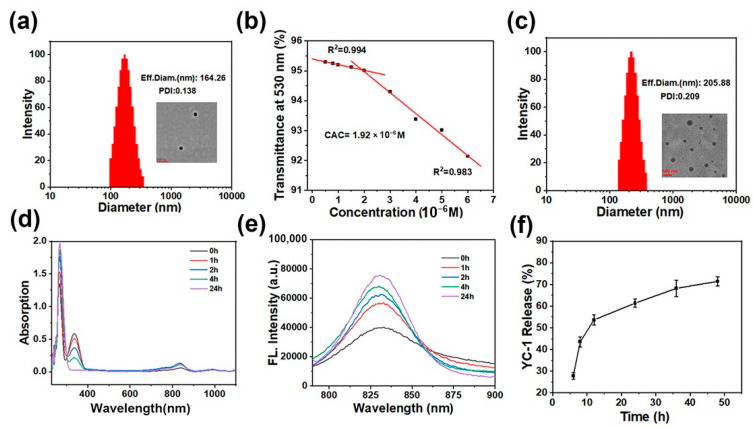
(**a**) DLS and TEM image of CyNPs; (**b**) Critical aggregation concentration (CAC) of CyNPs; (**c**) DLS and TEM image of GH@CyNPs; (**d**) Time-dependent changes in the UV-vis absorption spectra of CyNPs (10 μM) after incubation with rat liver microsomes and NADPH; (**e**) Time-dependent changes in the fluorescence spectra of CyNPs (10 μM) after incubation with rat liver microsomes and NADPH; (**f**) Percentage of YC-1 released from CyNPs upon incubation with rat liver microsomes and NADPH, mean ± SD (n = 3).

Driven by specific host–guest interactions, the mixed solution of **WP5** and **Cy-G** displayed a pronounced Tyndall effect, confirming successful nanoparticle assembly. Following stoichiometric optimization, a 1:4 molar ratio (**WP5**: **Cy-G**) was identified as optimal, yielding a robust colloidal system that remained stable for two weeks at ambient temperature. This nanoparticle was denoted as CyNPs. DLS analysis indicated a hydrodynamic diameter of 164 nm with a polydispersity index (PDI) of 0.138, and TEM images revealed that CyNPs exhibit a uniform spherical morphology with an average size of approximately 165 nm (Figure 1a). ζ-potential and critical aggregation concentration (CAC) of CyNPs were −23.7 mV and 1.92 × 10^−6^ M, respectively (Figure S13 and Figure 1b). Furthermore, GOx and CAT were co-encapsulated into CyNPs, endowing CyNPs with cascade catalytic activity via oxidation of the high-concentration glucose present in cancer cells to continuous release of O_2_. After removing the unloaded substances by dialysis, we successfully constructed the GOx and CAT co-loaded nanoplatform, termed GH@CyNPs. The hydrated diameter of GH@CyNPs was 206 nm, and the PDI was 0.209. TEM images showed a size of approximately 170~215 nm (Figure 1c). ζ-potential was −26.5 mV (Figure S14). The encapsulation efficiencies of GOx and CAT were determined to be approximately 47.5% and 37.0%, with corresponding drug loading contents of about 29.37% and 24.46%, respectively.

### 2.2. Hypoxia Responsiveness and Singlet Oxygen Generation Ability

To verify the hypoxia responsiveness, CyNPs were co-incubated with rat liver microsomes and NADPH to mimic the hypoxic tumor microenvironment. TEM images revealed that the spherical CyNPs gradually degraded as the incubation time increased. (Figure S15), confirming their hypoxia-responsive property. Moreover, the characteristic absorption peak of azo bonds at 350 nm gradually disappeared, accompanied by a marked increase in fluorescence intensity at 830 nm (Figure 1d,e), which collectively indicated the cleavage of azo bonds and subsequent photosensitizer cyanine release. Furthermore, the HIF-1α inhibitor YC-1 was also released under hypoxia, in which a cumulative YC-1 release rate of 71.43% was observed within 50 h (Figure 1f), demonstrating efficient hypoxia-triggered cargo release capability.

**Figure 2:**
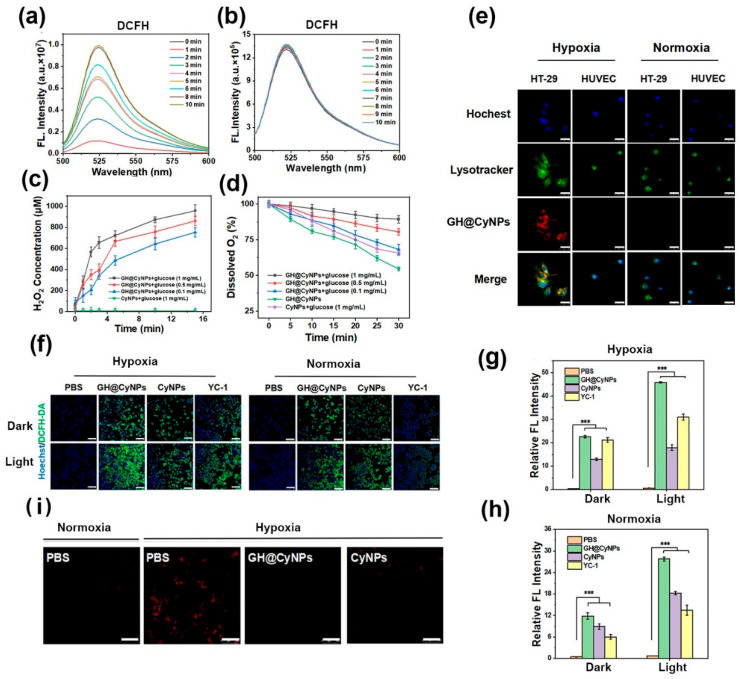
(**a**,**b**) ROS generation ability of CyNPs under normoxic and hypoxic conditions upon red-light irradiation; (**c**) H_2_O_2_ generation; (**d**) dissolved oxygen content change at different glucose concentrations and time points; (**e**) Cellular uptake imaging HT-29 or HUVEC cells after 2 h treatment by GH@CyNPs, scale bars: 25 μm; (**f**) ROS generation in HT-29 cells detected by the DCFH-DA probe under normoxic and hypoxic conditions, scale bars: 100 μm; (**g**,**h**) the corresponding quantitative analysis; (**i**) Intracellular O_2_ level imaging determined by O_2_ probe RTDP under different conditions, scale bars: 100 μm. * *p* < 0.05, ** *p* < 0.01, *** *p* < 0.001.

The ROS-generating capacity of hypoxia-degraded CyNPs under light irradiation was assessed using DCFH as a probe. After incubation of CyNPs in hypoxic conditions, the fluorescence intensity at 525 nm increased continuously with prolonged irradiation time (Figure 2a), whereas only negligible fluorescence was detected under normoxic conditions with the same light treatment (Figure 2b). The efficient production of singlet oxygen (^1^O_2_) was only detected in the hypoxia-degraded CyNPs group (Figure S16). These results indicate that hypoxia-degraded CyNPs can be effectively activated by light to execute PDT.

### 2.3. Glucose-Triggered Cascade Reaction

GOx catalyzes the oxidation of glucose to produce gluconic acid (H^+^) and H_2_O_2_, and the latter is subsequently decomposed by CAT to generate O_2_ [31,32]. This cascade reaction is crucial for alleviating hypoxia. Therefore, we first measured pH changes and H_2_O_2_ production of CyNPs and GH@CyNPs upon the addition of glucose. In contrast, only GH@CyNPs exhibited a significant pH decrease and obvious H_2_O_2_ production in the presence of glucose (Figure S17 and Figure 2c), with these changes positively correlated with glucose concentration. Subsequently, dissolved oxygen measurements revealed that the GH@CyNPs group exhibited the highest O_2_ content (approximately 89.34%, Figure 2d). Collectively, GH@CyNPs can efficiently oxidize glucose to produce O_2_ via cascade reactions, thereby alleviating hypoxia.

### 2.4. Cellular Uptake and Intracellular Generation of ROS and O_2_

To evaluate the cellular internalization of GH@CyNPs, HT-29 cancer cells and HUVEC normal cells were selected. As shown in Figure 2e, after 2 h of co-incubation under hypoxic conditions, GH@CyNPs-treated cancer cells exhibited obvious yellow fluorescence signals, indicating efficient internalization by cancer cells via endocytosis. In sharp contrast, negligible red fluorescence was detected in HUVEC normal cells and HT-29 cells cultured under normoxic conditions. These results confirm that GH@CyNPs possess both cancer cell-selective uptake and hypoxia-specific activation, enabling fluorescence signal production exclusively in the hypoxic tumor microenvironment.

The intracellular ROS level was evaluated using the ROS-specific fluorescent probe DCFH-DA. Under hypoxic conditions, the GH@CyNPs group displayed the highest fluorescence intensity (Figure 2f,h), reflecting robust ROS production. We deduced that this phenomenon is closely related to O_2_ levels in cancer cells. Thus, RTDP was utilized as a probe to quantify the intracellular oxygen content. Negligible RTDP fluorescence was observed in PBS-treated HT-29 cells under normoxic conditions (Figure 2i). In contrast, distinct red RTDP fluorescence was detected in PBS-treated HT-29 cells under hypoxic conditions, which is attributed to the quenching effect of O_2_ on RTDP fluorescence. Notably, no discernible RTDP fluorescence was observed in GH@CyNPs-treated cells under hypoxic conditions, comparable to that in PBS-treated cells under normoxic conditions. These results demonstrate that GH@CyNPs elevate intracellular oxygen levels under hypoxia, boosting ROS production and confirming their potent hypoxia-relieving capability.

### 2.5. In Vitro Therapeutic Effect

**Figure 3:**
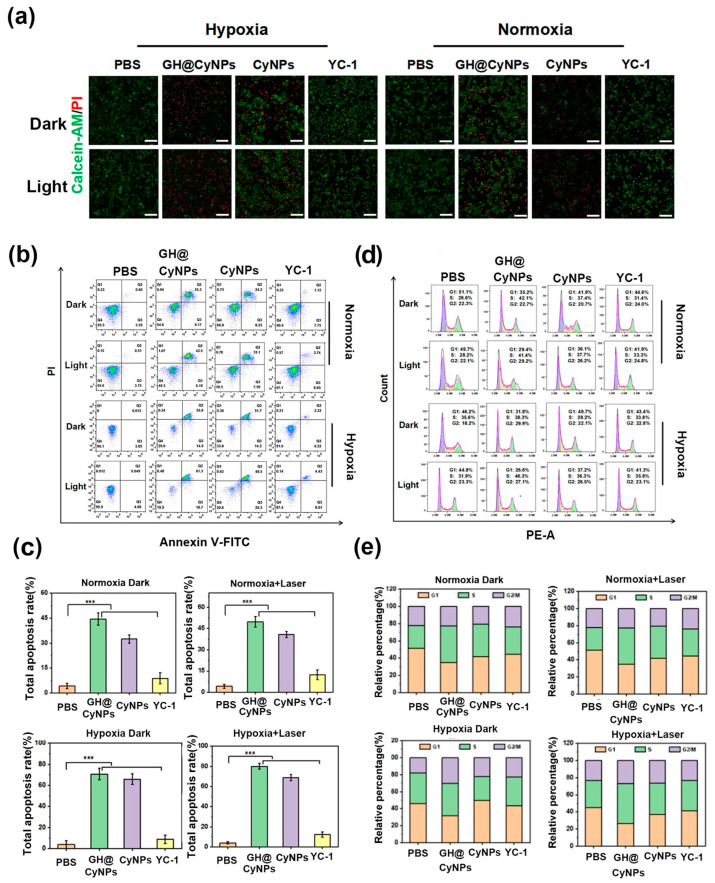
(**a**) Calcein-AM and PI staining of HT-29 cells after various treatments, scale bars: 100 μm; (**b**) Cell apoptosis and (**c**) apoptotic percentage analysis after various treatments by flow cytometry; (**d**) Cell cycle distribution and (**e**) quantitative analysis after various treatments by flow cytometry. * *p* < 0.05, ** *p* < 0.01, *** *p* < 0.001.

The cytotoxicity of GH@CyNPs against HT-29, MDA-MB-231, 4T1, and HUVEC cell lines was evaluated using MTT assays. GH@CyNPs had the lowest IC_50_ values against cancer cells compared to CyNPs and free YC-1, and its cytotoxicity can be reinforced by light irradiation (Table S1). Live/dead cell staining further validated the anticancer cell performance of GH@CyNPs (Figure 3a). Under hypoxic conditions with light irradiation, the GH@CyNPs group exhibited the highest proportion of dead cells (red fluorescence), indicating the most potent therapeutic cytotoxicity. This trend was in good agreement with the MTT cell viability assay results.

To further investigate the apoptotic induction effect on tumor cells, flow cytometry was applied to quantify cellular apoptosis levels. Under normoxic conditions, both CyNPs and YC-1 exhibited only a weak pro-apoptotic effect regardless of light irradiation. In contrast, the GH@CyNPs group presented a markedly elevated total apoptosis rate under normoxia, reaching 49.68% (dark) and 44.47% (light), respectively (Figure 3b,c). Under hypoxic conditions with light irradiation, the apoptotic effect of GH@CyNPs was further strengthened, with the total apoptosis rate up to 80%. These results demonstrate that the combination of hypoxia and light irradiation can remarkably enhance the apoptotic and cytotoxic efficacy of GH@CyNPs. To further clarify the underlying mechanism of GH@CyNPs-induced apoptosis, cell cycle distribution analysis was subsequently performed. The results revealed that GH@CyNPs could significantly arrest the tumor cell cycle at the S and G2 phases (Figure 3d,e). Such cell cycle arrest directly suppresses tumor cell proliferation and promotes downstream apoptotic signaling.

### 2.6. Cytotoxic Mechanisms

**Figure 4:**
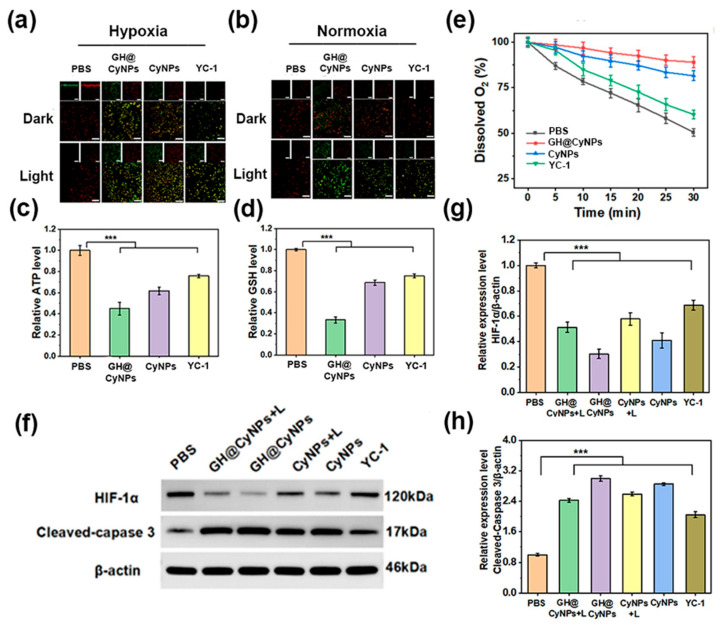
(**a**,**b**) JC-1 staining for mitochondrial membrane potential change, Scale bar = 100 μm; (**c**) The relative ATP content and (**d**) GSH level after different administrations; (**e**) Dissolved O_2_ content in culture medium containing HT-29 cells after treatment by various agents; (**f**) Measurement of HIF-1α and cleaved-caspase 3 expression level and (**g**,**h**) quantitative analysis after different administrations by Western blotting. * *p* < 0.05, ** *p* < 0.01, *** *p* < 0.001.

Excessive intracellular ROS generally disrupts the mitochondrial membrane potential, impairs mitochondrial function, and ultimately triggers cell death [33,34]. Accordingly, JC-1 staining was used to evaluate alterations in mitochondrial membrane potential following treatment (Figure 4a,b). As control groups, HT-29 cells incubated with PBS or YC-1 displayed negligible J-monomer signals under both normoxic and hypoxic conditions, irrespective of light irradiation, suggesting minimal mitochondrial damage. In comparison, both GH@CyNPs and CyNPs effectively reduced mitochondrial membrane potential and induced mitochondrial dysfunction, especially in irradiation. Quantitative analysis confirmed that GH@CyNPs possessed the most potent ability to disrupt mitochondrial function (Figure S18), which could be primarily attributed to robust intracellular ROS generation.

GOx can specifically consume intracellular glucose, thereby effectively blocking the ATP biosynthesis pathway at the initial stage of energy metabolism [35,36], starving cancer cells to death. Meanwhile, mitochondria serve as the primary site for cellular aerobic respiration; structural and functional damage to mitochondria hinders ATP synthesis via the oxidative phosphorylation (OXPHOS) pathway [37,38]. Together, these two pathways synergistically regulate intracellular ATP homeostasis in tumor cells. On this basis, we further examined the intracellular ATP levels under different treatments. In the GH@CyNPs group, ATP levels were significantly reduced to 44.9%, indicating a stronger energy inhibitory effect than that observed in the other groups (Figure 4c), which is attributed to the synergistic effect of GOx-mediated glucose depletion and ROS-induced cellular damage in the GH@CyNPs. Furthermore, GH@CyNPs could efficiently consume the excessively overexpressed GSH in tumor cells (Figure 4d). Marked depletion of GSH significantly impairs the antioxidant defenses of tumor cells, thereby boosting overall antitumor efficacy [39,40].

Next, the dissolved oxygen content was monitored in sealed HT-29 cell culture medium to investigate the alleviation of hypoxia by GH@CyNPs. After 30 min of co-incubation, the oxygen level in the PBS group decreased markedly to approximately 40~50% due to inherent cellular respiration. In contrast, the GH@CyNPs, CyNPs, and YC-1 groups all effectively suppressed cellular oxygen consumption (Figure 4e). Notably, the GH@CyNPs group maintained the highest oxygen level at around 89% under hypoxic conditions, resulting from its dual effects of GOx/CAT and YC-1. To further clarify the hypoxia-alleviating capacity of GH@CyNPs, Western blot (WB) analysis was performed to detect HIF-1α expression (Figure 4f–h). WB results confirmed that GH@CyNPs exerted the most pronounced downregulation of HIF-1α, demonstrating its excellent ability to relieve tumor hypoxia. Meanwhile, the GH@CyNPs group exhibited the highest expression of cleaved caspase-3, a canonical biomarker of cellular apoptosis. Notably, these two alterations were observed simultaneously in the same treatment group, reflecting a correlative relationship rather than a verified direct causal cascade [22].

### 2.7. In Vivo Distribution and Tumor Targeting

**Figure 5:**
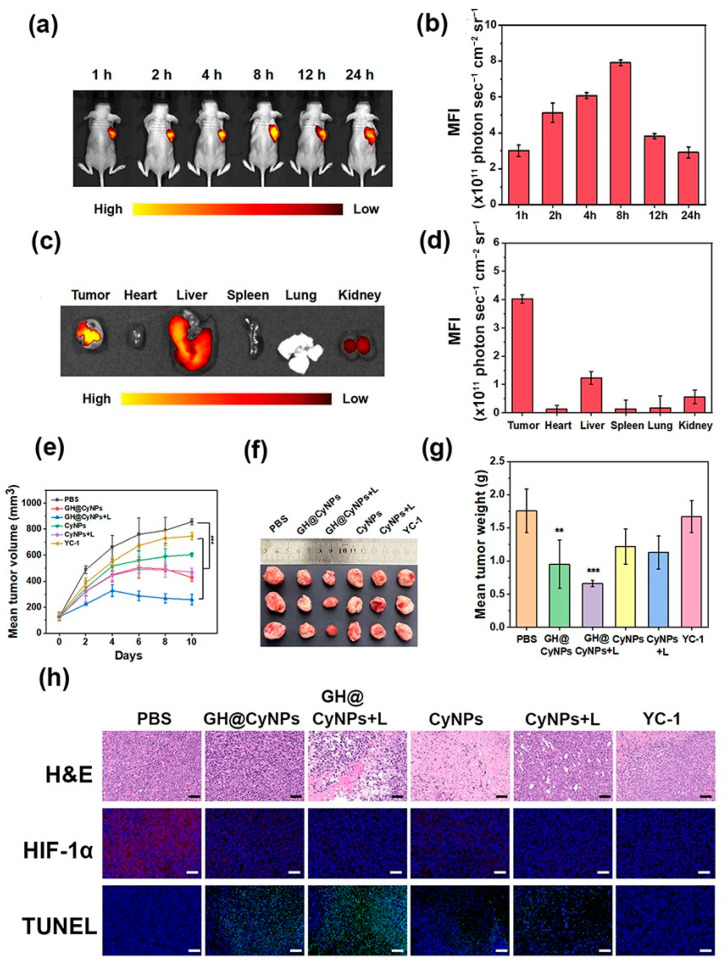
(**a**) In vivo distribution and accumulation of GH@CyNPs in HT-29 tumor-bearing mice post-injection intravenously and (**b**) quantitative analysis of MFI in the tumor area at different times; (**c**) Ex vivo imaging of major tissues and (**d**) quantitative analysis of MFI after 24 h injection; (**e**) Tumor volume change curve during 10 d treatment; (**f**) Dissected tumor graph and (**g**) weight after 10 d treatment; (**h**) Body weight of mice during 10 d treatment, Scale bar = 50 μm. * *p* < 0.05, ** *p* < 0.01, *** *p* < 0.001.

HT-29 tumor-bearing nude mice were used to evaluate the in vivo tissue distribution and tumor accumulation of GH@CyNPs. At 1 h post-injection, cyanine-derived fluorescence was detected in tumors and increased rapidly. The fluorescence signal gradually weakened at 8 h due to metabolic clearance but remained detectable at 24 h (Figure 5a,b). To further analyze the biodistribution of GH@CyNPs, tissues were dissected and imaged at 24 h post-injection. Ex vivo imaging revealed that cyanine fluorescence intensity in tumors was significantly higher than that in other tissues (Figure 5c). Quantitative analysis confirmed that GH@CyNPs exhibited excellent tumor accumulation (Figure 5d), highlighting its potential for in vivo tumor therapy. Additionally, these results indicated that azo bonds in GH@CyNPs could be cleaved in the hypoxic tumor microenvironment, and the released Cy could serve for tumor diagnosis.

### 2.8. In Vivo Antitumor Performance and Biocompatibility of GH@CyNPs

Encouraged by the outstanding in vitro growth inhibition and in vivo biodistribution, we further evaluated the therapeutic potential of GH@CyNPs using an HT-29 tumor-bearing xenograft mouse model. As shown in Figure 5e, the PBS-treated group exhibited aggressive tumor progression, with volumes surging approximately six-fold over the 10-day treatment. In sharp contrast, the light-irradiated GH@CyNPs group exerted an outstanding inhibitory effect on tumor progression. This superior performance was attributed to its efficient tumor targeting and accumulation, coupled with synergistic therapeutic effects via intracellular cascade reactions. Notably, the non-irradiated GH@CyNPs group exhibited significantly attenuated in vivo antitumor activity, as evidenced by a persistent increase in tumor volume. This distinct difference demonstrates that PDT plays a pivotal role in tumor growth inhibition. After 10 days of treatment, tumor-bearing mice were euthanized, and tumor tissues were dissected for weighing, photography, and further analysis. The light-irradiated GH@CyNPs group showed the most prominent inhibition of tumor growth and achieved the optimal reduction in tumor weight among all groups (Figure 5f,g). Moreover, the body weight of mice in each group remained stable without abnormal fluctuations throughout the treatment period. These results confirm that GH@CyNPs possess potent in vivo antitumor activity. Histological evaluation of dissected tumor tissues, comprising H&E staining, HIF-1α immunofluorescence staining, and TUNEL staining, was conducted to further assess antitumor efficacy. Compared with other groups, the GH@CyNPs with light irradiation group showed the most remarkable effects on tumor cell damage, HIF-1α downregulation, and apoptosis induction (Figure 5h).

Moreover, H&E staining results showed no obvious pathological injury or abnormal morphological changes in major organs of the GH@CyNPs group compared with the PBS group (Figure S19a). To further systematically assess in vivo biosafety, healthy mice were intravenously administered with GH@CyNPs. Biochemical analysis related to liver function (ALT, AST) and renal function (BUN, CRE), as well as routine blood parameters, were subsequently examined. All detected biomarkers remained within the normal physiological range without significant alterations (Figure S19b). Furthermore, the in vitro hemolysis assay indicated that GH@CyNPs exhibited negligible hemolytic activity, with a hemolysis rate below 1% (Figure S19c). Together, these results indicate that the GH@CyNPs possess a favorable antitumor efficacy and a good biosafety profile.

## 3. Materials and Methods

### 3.1. Preparation of CyNPs and GH@CyNPs

CyNPs: **WP5** and **Cy-G** were dissolved in water and absolute ethanol, respectively, to obtain **WP5** stock solution (2 × 10^−3^ M) and **Cy-G** stock solution (1 × 10^−2^ M). Subsequently, **WP5** (62.5 μL) and **Cy-G** derivative (50 μL) were sequentially injected into water to prepare a 5 mL solution. After shaking evenly for several minutes, stable supramolecular nanoparticles were immediately obtained and designated as CyNPs.

GH@CyNPs: GOx (10 mg/mL, dissolved in water, 100 μL), CAT (2 mg/mL, dissolved in water, 500 μL), WP5 (62.5 μL), and Cy-G (50 μL) were added to water in sequence to form a 5 mL solution. The mixture was then stored overnight in the dark and dialyzed to remove unloaded GOx, CAT, and organic solvents (molecular weight cutoff = 300,000). Afterwards, GH@CyNPs were prepared. Calculation of GOx/CAT loading capacity: The absorbance at 562 nm was measured by loading GOx or CAT separately. The protein concentration was calculated according to the standard curve of protein standards (concentration range: 0 to 500 μg/mL), and the loading capacity was further computed.

### 3.2. Hypoxic Response of CyNPs

An aqueous solution of CyNPs (10 μM) containing rat liver microsomes (15 μL, 20 mg/mL) (Shanghai Biolu Biotechnology Co., Ltd., Shanghai, China) and nicotinamide adenine dinucleotide phosphate disodium salt (NADPH) (1 mM) was incubated at 37 °C under hypoxic conditions (1% O_2_, 5% CO_2_, 94% N_2_). The hypoxia-induced degradation of CyNPs at different time points was evaluated using an ultraviolet spectrophotometer (Shanghai Jinghua Science & Technology Instrument Co., Ltd., Shanghai, China) and a fluorescence spectrophotometer (Shimadzu Corporation, Kyoto, Japan).

### 3.3. Detection of Extracellular Reactive Oxygen Species (ROS)

DCFH (2′,7′-dichlorodihydrofluorescein) was selected as the ROS indicator, and deionized water was used as the solvent to prepare the relevant system throughout the experiment. DCFH (10 μM) was added to the aqueous solution of CyNPs (100 μM). After mixing evenly, the mixture was irradiated with a 650 nm laser (30 mW/cm^2^). Subsequently, the change in fluorescence intensity was measured by a fluorescence spectrophotometer with an excitation wavelength of 488 nm and an emission wavelength of 525 nm. Data was recorded every 1 min of light irradiation for a continuous monitoring period of 10 min to evaluate the responsiveness of CyNPs to ROS.

### 3.4. In Vitro Detection of Singlet Oxygen (^1^O_2_)

DPBF (1,3-diphenylisobenzofuran) was selected as the singlet oxygen scavenger, and deionized water was used as the solvent to prepare the relevant system during the experiment. DPBF was added to CyNPs (100 μM), and the absorbance of DPBF at 412 nm was adjusted to approximately 1.0. Subsequently, the side of the quartz cuvette was irradiated with a 650 nm laser (30 mW/cm^2^) for a total irradiation time of 40 min. The absorption spectra were recorded at different time intervals within 40 min. The scanning wavelength range was set from 350 to 600 nm with a scanning rate of 1.0 nm/s.

### 3.5. pH Change Measurement

Glucose of different concentrations was added into GH@CyNPs (1.0 × 10^−4^ M) and CyNPs (1.0 × 10^−4^ M), and the pH values of the solutions were measured at different time points using a pH meter (Mettler Toledo, Zurich, Switzerland).

### 3.6. Hydrogen Peroxide (H_2_O_2_) Generation

TMB (0.1 mM/L) and HRP (1 mU/mL) were added to the solutions containing GH@CyNPs (1.0 × 10^−4^ M) and CyNPs (1.0 × 10^−4^ M), respectively. Subsequently, glucose at concentrations of 0.1, 0.5 or 1 mg/mL was added to the solutions at 37 °C. The absorbance at the wavelength of 652 nm was measured at different time points using an ultraviolet–visible spectrophotometer (Shanghai Jinghua Science & Technology Instrument Co., Ltd., Shanghai, China), and the concentration of hydrogen peroxide (H_2_O_2_) in the system was calculated according to the standard curve of H_2_O_2_.

### 3.7. Determination of Dissolved Oxygen (O_2_) Content In Vitro

Glucose (0.1, 0.5 or 1 mg/mL) was added to the solutions containing GH@CyNPs (1.0 × 10^−4^ M) and CyNPs (1.0 × 10^−4^ M), which were then sealed with liquid paraffin to exclude external oxygen supply. The dissolved oxygen content in the culture medium was measured at different time points using an oxygen content analyzer (Shanghai INESA Scientific Instrument Co., Ltd., Shanghai, China) for a duration of 30 min.

## 4. Conclusions

In this work, we have successfully engineered a hypoxia-activatable supramolecular nanoplatform, GH@CyNPs, designed to address the limitations of PDT in oxygen-deprived tumor microenvironments. GH@CyNPs represent a self-assembled nanosystem that integrates photosensitizers, HIF-1α inhibitors, and functional enzymes (GOx and CAT) via host–guest interactions. Under hypoxic conditions, GH@CyNPs can be effectively dissociated to release YC-1, GOx, and CAT, achieving dual hypoxia alleviation through the synergistic effect of HIF-1α inhibition and O_2_ generation. This synergistic effect profoundly amplifies ROS production upon light exposure, leading to efficient suppression of cancer cell growth and induction of apoptosis. In vivo evaluations further validated that GH@CyNPs exhibited remarkable tumor-specific accumulation, potent suppression of tumor growth, and good biosafety. By overcoming key barriers of the hypoxic TME, this study offers a valuable reference for developing safe, synergistic anticancer therapies against hypoxic tumors. However, it exhibits certain restrictions in long-term tumor therapy, particularly against deep hypoxic tumors.

## Data Availability

Data are contained within the article and Supplementary Materials.

## Supplementary Materials

The following supporting information can be downloaded at: https://www.mdpi.com/article/10.3390/molecules31142433/s1, General Information, Experimental procedure, Synthesis of Guest Molecule Cy-G, ζ-potentials of the CyNPs, ζ-potentials of the GH@CyNPs, TEM images of CyNPs under hypoxic conditions, Singlet oxygen generation capability of CyNPs, Glucose-induced pH changes, Cytotoxicity, Cytotoxic Mechanism, Biosafety, Raw data of Western blot.
