# Supplementary material for: Development of a Hypoxia-Triggered Supramolecular Nanoplatform for Synergistic Hypoxia Alleviation and Amplified Photodynamic Cancer Therapy
Source: Molecules. 2026 Jul 11;31(14):2433. doi: 10.3390/molecules31142433 (PMC13414287; doi:10.3390/molecules31142433)
Supplement: Supplementary file 1 [file molecules-31-02433-s001.zip › molecules-4401723-supplementary.pdf]

Supporting Information

Development of a Hypoxia-Triggered Supramolecular Nanoplatform for Synergistic Hypoxia Alleviation and Amplified Photodynamic Cancer Therapy

Ningning Luo[1, †], Yiliang Wu [1, †], Jiaxin Zheng[1], Chi Zhang[1], Xiaoyang Qian[1], Caoqing Ji[1], Aiqing Jiang[2], Yong Ling[1, *], Xin Liu[1, *]

1 Jiangsu Province Key Laboratory for Inflammation and Molecular Drug Target, School of Pharmacy, Nantong University, Nantong 226001, China
2 Jiangsu Key Laboratory of Molecular Medicine, Medical School of Nanjing University, Nanjing 210093, China
* Correspondence: lyyy111@sina.com (Y.L.); xinliunju@foxmail.com (X.L.)
† These authors contributed equally to this work.

Table of Contents

1. General Information 3

2. **Experimental procedure** 4

3. Synthesis of Guest Molecule Cy-G 10

4. ζ-potentials of the CyNPs 23

5. ζ-potentials of the GH@CyNPs. 23

6. TEM images of CyNPs under hypoxic conditions 24

7. Singlet oxygen generation capability of CyNPs 24

8. Glucose-induced pH changes 25

9. Cytotoxicity 25

10. Cytotoxic Mechanism 26

11. Biosafety 26

12. Raw data of Western blot 27

1. General Information

All chemical reagents and solvents used in this study were purchased from commercial sources. HT-29 cancer cells, MDA-MB-231 cancer cells, 4T1 cancer cells, and HUVEC normal cells were purchased from the National Collection of Authenticated Cell Cultures, Chinese Academy of Sciences. The progress of all reactions was monitored in real time by thin-layer chromatography, and the crude products obtained from the reactions were separated and purified by silica gel column chromatography. ^{1}H NMR and ^{13}C NMR spectra were recorded on a Bruker AV 400M nuclear magnetic resonance spectrometer, and high-resolution mass spectrometry data were acquired using an LC/MSD TOF mass spectrometer. The laser light adopted in the experiment was the LWRPD-200F model (Beijing Laser Wave). The purities of the final target products **WP5** and **Cy-G** were both higher than 95%, as determined by high-performance liquid chromatography. The main experimental instruments include: clean bench (Antai Co., Ltd., China), CO_2 incubator (SANYO, Japan), inverted biological microscope (OLYMPUS, Japan), tabletop low-speed centrifuge (Medical Equipment Factory of Shanghai Medical Instrument Co., Ltd.), micropipette (Eppendorf, Germany), microplate reader (BioTek, USA), vortex mixer (Haimen Qilinbell Instrument Manufacturing Co., Ltd.), electronic analytical balance (TOLEDO, USA), ultraviolet spectrophotometer (Shanghai Jinghua Technology Instrument Co., Ltd.), fluorescence spectrophotometer (SHIMADZU, Japan), ultrapure water system (Nanjing Oukai Environmental Technology Co., Ltd.), laser confocal fluorescence microscope (LEICA, Germany), and frozen section microtome (LEICA, Germany).

2. **Experimental procedure**

2.1 Cellular Uptake and Intracellular Distribution

HT-29 cells (human colon cancer cells) or HUVECs (human umbilical vein endothelial cells) were cultured for 24 hours before treatment. Subsequently, the cells were co-incubated with GH@CyNPs (10 μM) under normoxic conditions (20% O_2, 5% CO_2, and 75% N_2 at 37 °C) or hypoxic conditions (1% O_2, 5% CO_2, and 94% N_2 at 37 °C) for 2 hours. After incubation, the culture medium was discarded, and the cells were washed three times with PBS. Thereafter, lysosomes and cell nuclei were labeled with Lysotracker Green and Hoechst 33342 (Thermo Fisher, USA), respectively, following standard protocols. Intracellular fluorescence was monitored by CLSM.

2.2 Detection of intracellular reactive oxygen species (ROS)

Incubate HT-29 cells with PBS, GH@CyNPs (10 μM), CyNPs (10 μM) or YC-1 (10 μM) under normoxic conditions (20% O_2, 5% CO_2, and 75% N_2, 37 °C) or hypoxic conditions (1% O_2, 5% CO_2, and 94% N_2, 37 °C). After 4 hours, add the ROS probe DCFH-DA (10 μM) and Hoechst 33342 (5 μg/mL), and incubate for 30 minutes. The irradiation group was treated with NIR irradiation (650 nm, 30 mW/cm^2, 10 minutes), and the intracellular ROS generation was observed by CLSM.

2.3 Intracellular O_2 Level Imaging

HT-29 cells were seeded in culture dishes and incubated for 24 hours under normoxic conditions (20% O_2, 5% CO_2, and 75% N_2, 37 °C) or hypoxic conditions (1% O_2, 5% CO_2, and 94% N_2, 37 °C), respectively. Subsequently, the cells were treated with PBS, GH@CyNPs (10 μM), or CyNPs (10 μM) for 4 hours. Afterwards, the culture medium was replaced with fresh medium containing 10 μg/mL RTDP probe. After incubation for 6 hours, the cells were washed three times with PBS to remove unbound RTDP probe, followed by the addition of fresh medium, and imaging was performed via CLSM.

2.4 In Vitro Cytotoxicity

The relative in vitro cytotoxicity against HT-29 cancer cells, MDA-MB-231 cancer cells, 4T1 cancer cells, and HUVEC normal cells was evaluated via MTT assay. Briefly, the cells were treated with different groups (including GH@CyNPs, CyNPs and YC-1) at various concentrations under normoxic conditions (20% O_2, 5% CO_2 and 75% N_2, 37 °C) or hypoxic conditions (1% O_2, 5% CO_2 and 94% N_2, 37 °C) for 12 hours with or without irradiation (650 nm, 30 mW/cm^2, 10 min), followed by the MTT assay.

2.5 Live/Dead Cell Staining

HT-29 cells were incubated with PBS, GH@CyNPs (10 μM), CyNPs (10 μM), or YC-1 (10 μM) for 12 hours under normoxic conditions (20% O_2, 5% CO_2 and 75% N_2, 37 °C) or hypoxic conditions (1% O_2, 5% CO_2 and 94% N_2, 37 °C) with or without irradiation (650 nm, 30 mW/cm^2, 10 minutes). After staining with 2 μM calcein AM and 4 μM PI for 30 minutes, cell viability was observed by CLSM imaging.

2.6 Flow Cytometry Analysis

HT-29 cells were seeded in 12-well plates at a density of 1×10^5 cells per well and cultured in 1 mL of complete DMEM medium at 37 °C for 24 hours. Subsequently, cells were co-cultured with PBS, GH@CyNPs (10 μM), CyNPs (10 μM) or YC-1 (10 μM) for another 24 hours under normoxic conditions (20% O_2, 5% CO_2 and 75% N_2 at 37 °C) or hypoxic conditions (1% O_2, 5% CO_2 and 94% N_2 at 37 °C), with or without irradiation (650 nm, 30 mW/cm^2, 10 minutes). The cell cycle distribution was then analyzed using the Cell Cycle and Apoptosis Detection Kit (Beyotime, China), and the level of cell apoptosis was determined with the Annexin V-FITC Apoptosis Detection Kit (Beyotime, China). Relevant results of apoptosis and cell cycle were acquired by a BD FACS Calibur flow cytometer, with 10^4 cells detected for each sample.

2.7 Mitochondrial damage

HT-29 cells were incubated with PBS, GH@CyNPs (10 μM), CyNPs (10 μM) or YC-1 (10 μM), respectively, under normoxic conditions (20% O_2, 5% CO_2 and 75% N_2, 37 °C) or hypoxic conditions (1% O_2, 5% CO_2 and 94% N_2, 37 °C), with or without irradiation (650 nm, 30 mW/cm^2, 10 minutes). After 12 hours of incubation, JC-1 (5 μg/mL) was added for staining for 30 minutes, followed by imaging via CLSM.

2.8 Determination of Intracellular ATP Level

HT-29 cells were seeded in 6-well plates at a density of 8×10^4 cells per well and cultured routinely for 24 hours to allow adherent growth. After the cells reached an appropriate growth state, they were treated with PBS, GH@CyNPs (10 μM), CyNPs (10 μM), and YC-1 (10 μM), respectively, for 6 hours. Upon completion of the treatment, the intracellular ATP level was determined in accordance with the recommended operating procedures of the Beyotime (China) ATP Assay Kit.

2.9 Determination of Intracellular Glutathione (GSH)

HT-29 cells were seeded in 6-well plates at a density of 5×10^4 cells per well and cultured for 24 hours. Subsequently, the cells were treated with PBS, GH@CyNPs (10 μM), CyNPs (10 μM), or YC-1 (10 μM), respectively, for 24 hours. Afterwards, the cells were washed three times with PBS, and the intracellular GSH content was determined using a Total GSH Assay Kit (Beyotime, China) without the addition of glutathione reductase.

2.10 Determination of Dissolved Oxygen (O_2) Content

The 4T1 cells were incubated with PBS, GH@CyNPs (10 μM), CyNPs (10 μM), or YC-1 (10 μM), respectively, under normoxic conditions (20% O_2, 5% CO_2, 75% N_2, 37 °C) or hypoxic conditions (1% O_2, 5% CO_2, 94% N_2, 37 °C) for 8 hours. The culture medium was sealed with liquid paraffin to exclude external oxygen supply. The

dissolved oxygen content in the culture medium was measured using an oxygen content analyzer at different time points for a duration of 30 minutes continuously.

2.11 Western Blot analysis

HT-29 cells were seeded in 6-well plates at a density of 5×10^6 cells per well and incubated at 37 °C for 24 hours. After the cells adhered and grew to an appropriate state, they were transferred to a hypoxic culture environment (1% O_2, 5% CO_2, 94% N_2, 37 °C). The cells were treated with PBS, GH@CyNPs (10 μM), CyNPs (10 μM), and YC-1 (10 μM), respectively, for 12 hours. During the treatment, an irradiation group (650 nm, 30 mW/cm^2, irradiation for 10 minutes) and a non-irradiation group were set as controls. After treatment, total cellular protein was extracted following standard experimental procedures, and a Western blot assay was used to analyze protein expression, with β-actin serving as the internal reference protein to calibrate the experimental results.

2.12 Establishment of Mouse Tumor Model

All animal experimental protocols were reviewed and approved by the Animal Research and Care Committee of Nantong University (No. S20210925-003) to ensure that the experimental procedures complied with animal ethical requirements. To establish the HT-29 tumor model, female BALB/c nude mice aged 4–5 weeks were purchased from the Laboratory Animal Center of Nantong University in this study. A total of 1×10^6 HT-29 tumor cells were inoculated into the nude mice via subcutaneous injection. The mice were raised under routine conditions, and tumor growth was observed. When the tumor volume in the nude mice reached approximately 100 mm^3, relevant in vivo experiments were officially initiated.

2.13 In vivo and in vitro fluorescence imaging

Sterilized GH@CyNPs nano formulation (200 μM, 200 μL) was injected into HT-

29 tumor-bearing nude mice via the tail vein. At six time points of 1, 2, 4, 8, 12, and 24 hours after injection, the mice were fully anesthetized, and then in vivo fluorescence imaging detection was performed using the Tanon ABL series in vivo imaging system. To further explore the in vivo biodistribution characteristics of GH@CyNPs, the mice were sacrificed 24 hours after injection. Their major organs (heart, liver, spleen, lung, and kidney) and tumor tissues were dissected for ex vivo fluorescence imaging analysis. Meanwhile, the fluorescence intensity of the region of interest (ROI) was quantitatively analyzed with the help of Tanon ABL series in vivo imaging software.

2.14 In vivo tumor suppression

A total of 200 μL of PBS, GH@CyNPs (200 μM), CyNPs (200 μM) or YC-1 (200 μM) were intravenously injected into HT-29 tumor-bearing mice every two days (3 mice per group), and the treatment was performed with or without NIR irradiation (650 nm, 30 mW/cm^2, 10 minutes). During the entire treatment period, the body weight and tumor volume of the mice were measured and recorded every two days. The calculation formula for tumor volume (V) is: width^2 × length / 2. After 10 days of treatment, mice in all groups were sacrificed, and major organs and tumors were harvested for H&E, HIF-1α, and TUNEL staining analysis.

2.15 Blood Biochemical Test

A total of 200 μL of PBS or GH@CyNPs (200 μM) were intravenously injected into healthy mice every three days (3 mice per group). After 15 days of treatment, mice in all groups were sacrificed, and blood samples were collected for blood biochemical analysis.

2.16 Hemolysis test

Erythrocytes (RBCs) were isolated from fresh blood of healthy male ICR mice and centrifuged at 3000 rpm for 5 minutes. Subsequently, the erythrocytes were washed three times with PBS buffer until the supernatant became colorless. The erythrocytes were then suspended in 49 mL of PBS (10 mM, pH 7.4). Next, 0.50 mL of erythrocyte

suspension was mixed with 0.50 mL of PBS buffer containing different concentrations of GH@CyNPs, with final concentrations of 5, 7.5, 10 and 15 μM, respectively. For the positive control and negative control, 0.50 mL of erythrocyte suspension was mixed with 0.50 mL of deionized water or PBS, respectively. After mixing well, the mixture was incubated at room temperature for 2 h, followed by centrifugation at 3000 rpm for 5 min. The absorbance of the supernatant was measured at 540 nm using an ultraviolet-visible spectrophotometer, and the hemolysis percentage was calculated according to the following formula:

$$\text{Hemolysis (\%)} = (A_s - A_{nc}) / (A_{pc} - A_{nc}) * 100$$

Among them, As represents the absorbance of the sample; Apc represents the absorbance of the positive control; and Anc represents the absorbance of the negative control.

2.17 Statistical Analysis

Statistical analysis of different groups was performed using Student's T-test. In all figures, error bars represent mean ± standard deviation (SD) ($n \geq 3$ independent experiments unless otherwise specified). P-values are indicated in the figure panels as $^*P < 0.05$ (significant), $^{**}P < 0.01$ (moderately significant), and $^{***}P < 0.001$ (highly significant).

3. Synthesis of Guest Molecule Cy-G

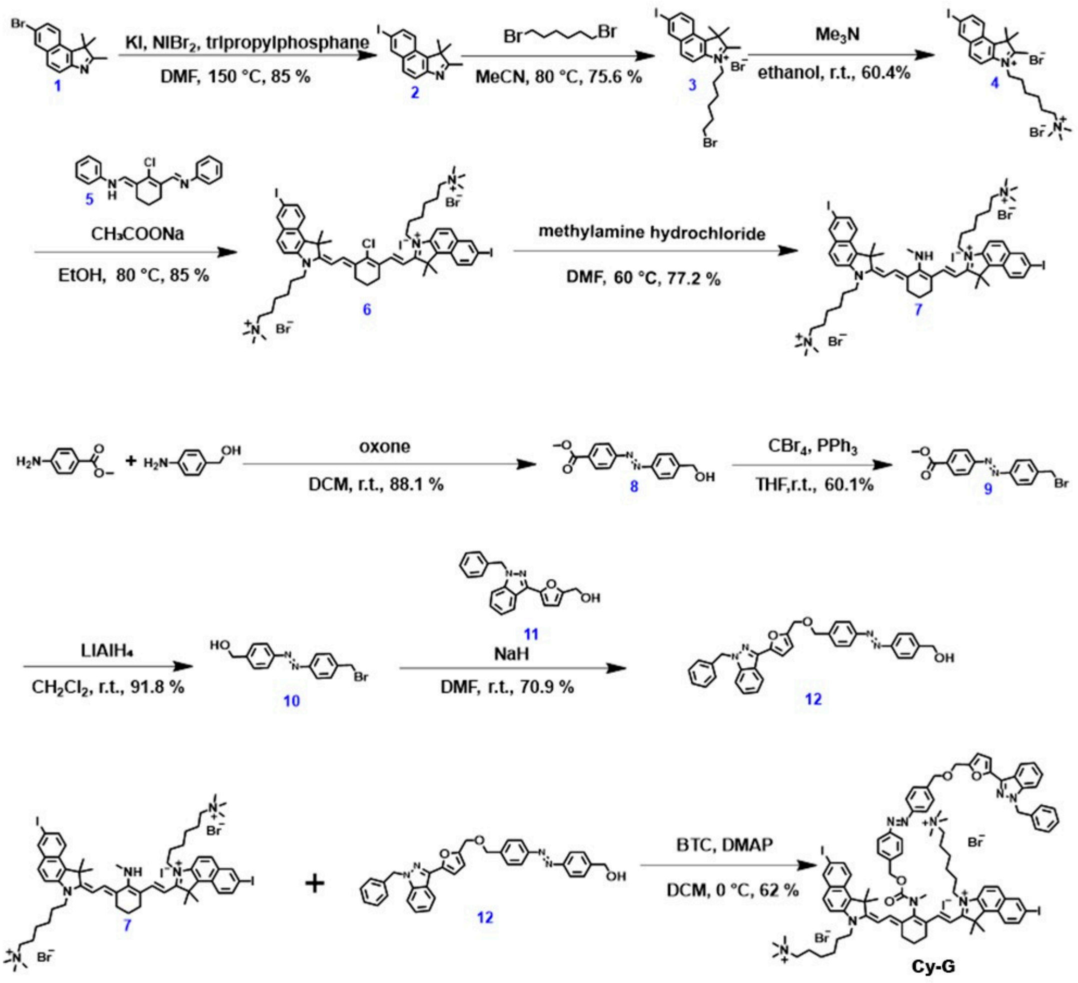

Scheme S1. Synthetic route of master **Cy-G**.

Synthesis of Compound **2**

To a 30 mL DMF solution containing 7-bromo-1,1,2-trimethyl-1H-benzo[e]indole (2.87 g, 10 mmol), KI (8.3 g, 50 mmol) and $NiBr_2$ (218 mg, 1 mmol), tripropylphosphine (202 mg, 1 mmol) was added. The reaction was carried out at 150 °C under nitrogen protection and monitored by TLC until completion. The reaction mixture was poured into 200 mL of ice water and extracted with ethyl acetate. The organic extracts were combined, washed with brine, dried over anhydrous sodium sulfate and concentrated by rotary evaporation. The residue was purified by column chromatography (EA/PE = 1/8) to afford compound **2** as a brown solid (2.85 g, 85%

yield). ^1H NMR (400 MHz, DMSO-d_6) δ 8.42 - 8.37 (m, 1H), 8.32 (d, J = 8.9 Hz, 1H), 8.26 - 8.21 (m, 1H), 8.15 (d, J = 8.9 Hz, 1H), 7.81 (m, J = 8.2, 6.8, 1.5 Hz, 1H), 4.15 (s, 3H), 1.79 (s, 6H).

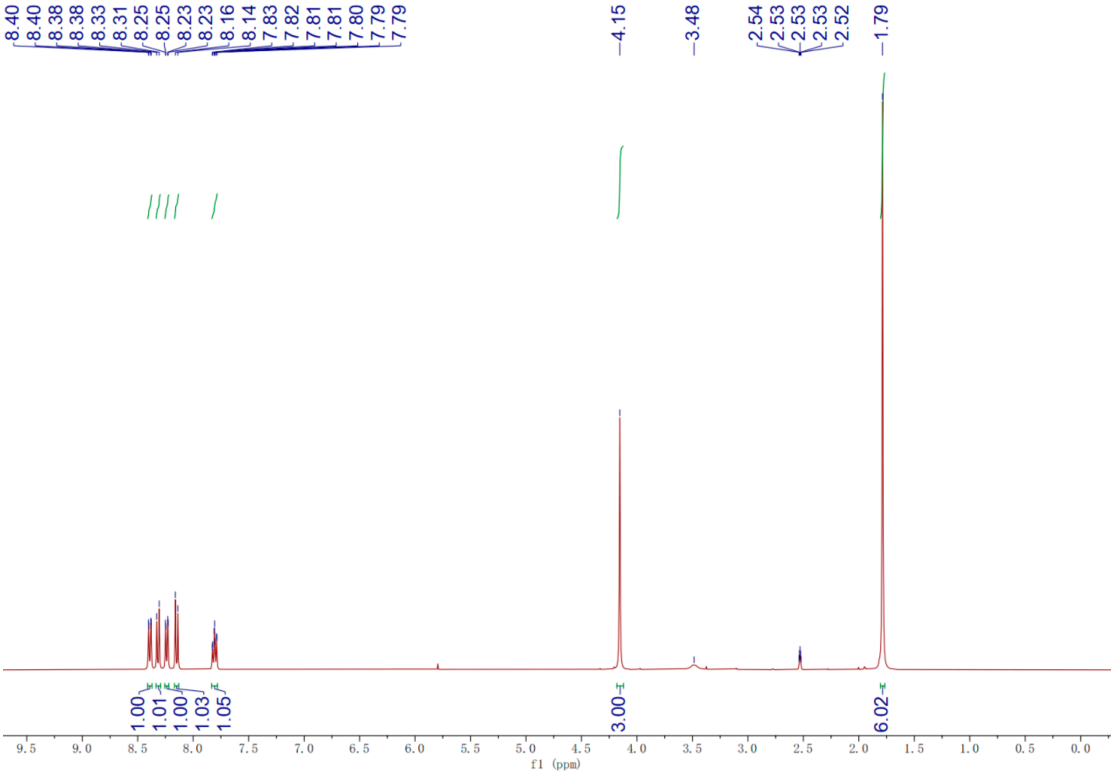

Figure S1. ^1H NMR spectrum (400 MHz, DMSO-d_6, 298 K) of compound **2**.

Synthesis of Compound **3**

Compound **2** (3.35 g, 10 mmol) and 1,6-dibromohexane (4.98 g, 20 mmol) were dissolved in 50 mL of acetonitrile, and the mixture was reacted at 80 °C under nitrogen protection. The reaction was monitored by TLC until completion. After the reaction was finished, the solvent was removed under reduced pressure to afford compound **3** as a brown solid (4.38 g, 75.6%). ^1H NMR (400 MHz, DMSO-d_6) δ 8.73 (d, J = 1.8 Hz, 1H), 8.25 (d, J = 9.0 Hz, 1H), 8.22 – 8.18 (m, 2H), 8.01 (dd, J = 8.8, 1.8 Hz, 1H), 4.56 (t, J = 7.3 Hz, 2H), 3.54 (d, J = 6.9 Hz, 2H), 2.94 (s, 3H), 1.93 - 1.86 (m, 2H), 1.85 - 1.76 (m, 2H), 1.75 (s, 6H), 1.53 - 1.37 (m, 4H).

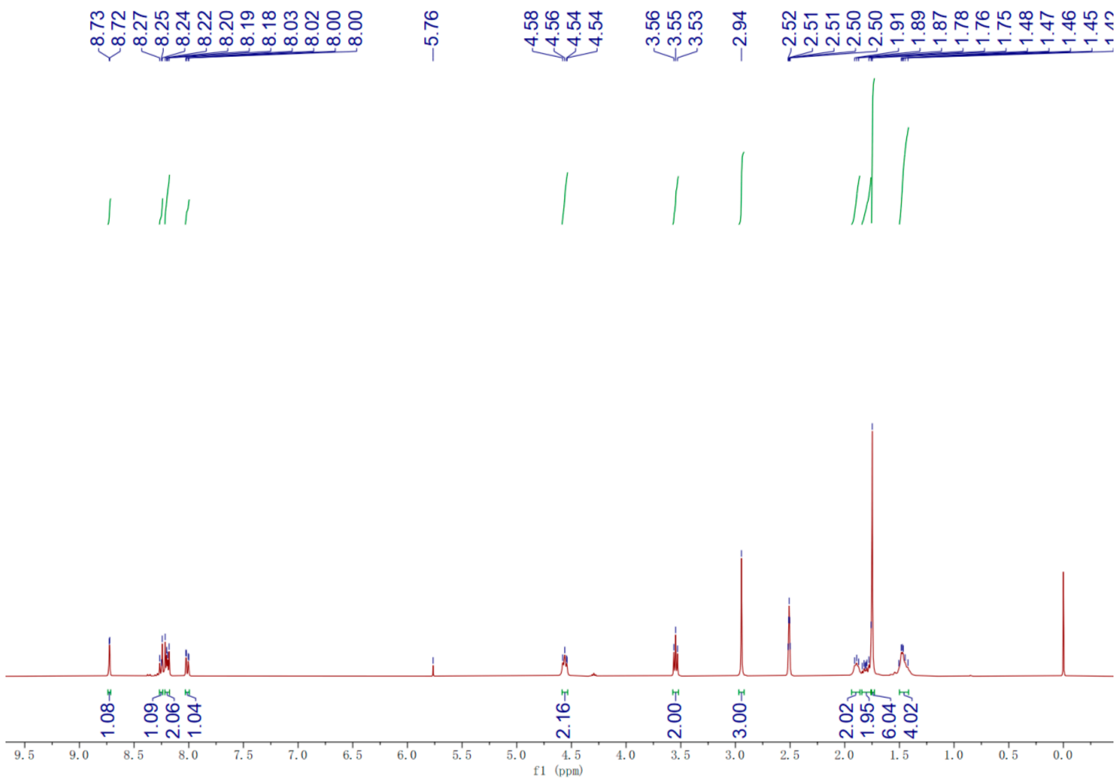

Figure S2. ^{1}H NMR spectrum (400 MHz, DMSO-d_6, 298 K) of compound **3**.

Synthesis of Compound **4**

Compound **3** (4.49 g, 8.4 mmol) was mixed with an ethanol solution of trimethylamine (4.2 M, 2.2 mL, 9.2 mmol) and reacted at room temperature for 48 hours. Upon completion of the reaction, the solvent was removed under reduced pressure, and the solid was washed several times with ethyl acetate to afford Compound **4** as a brown solid (3.24 g, 60.4%).^{1}H NMR (400 MHz, DMSO-d_6) δ 8.73 (d, J = 1.8 Hz, 1H), 8.26 (d, J = 9.0 Hz, 1H), 8.22 (d, J = 5.0 Hz, 1H), 8.19 (d, J = 4.8 Hz, 1H), 8.02 (dd, J = 8.9, 1.8 Hz, 1H), 4.58 (t, J = 7.5 Hz, 2H), 3.05 (s, 9H), 2.96 (s, 3H), 1.76 (s, 6H), 1.72 - 1.64 (m, 2H), 1.54 -1.47 (m, 2H), 1.35 (m, J = 8.4 Hz, 4H).

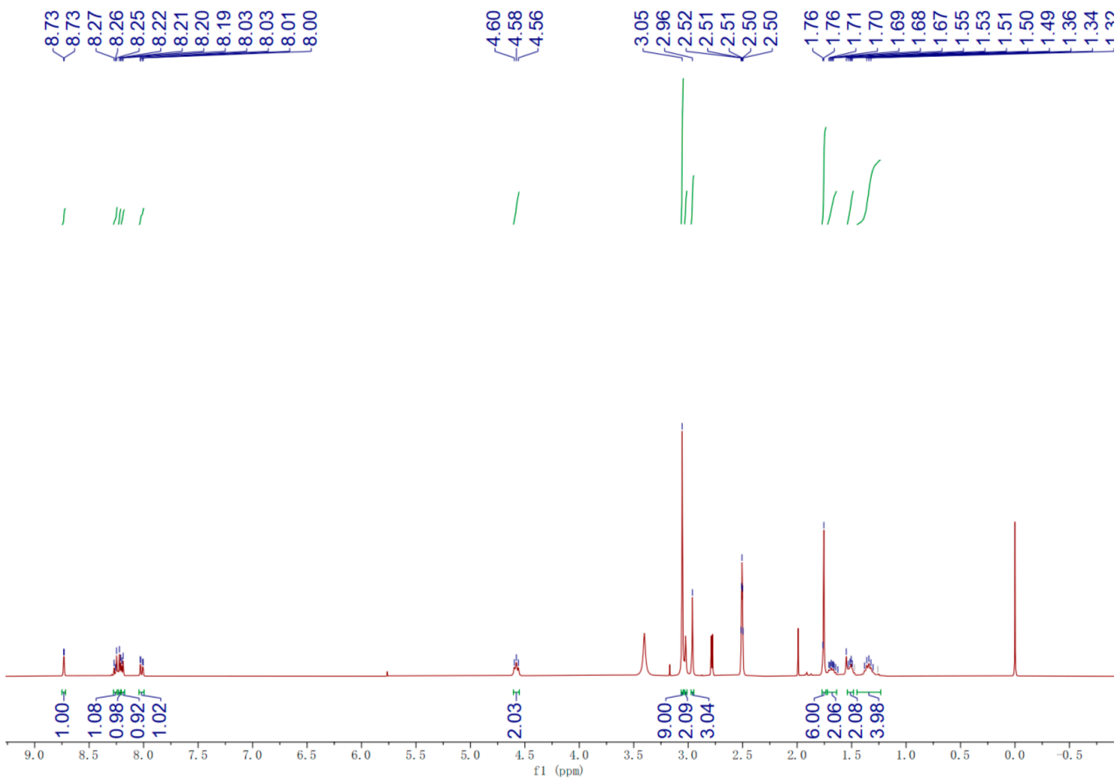

Figure S3. ^{1}H NMR spectrum (400 MHz, DMSO-d_6, 298 K) of compound **4**.

Synthesis of Compound **6**

Dissolve Compound **4** (6.36 g, 10 mmol), Compound 5 (1.62 g, 5 mmol) and CH_3COONa (1.23 g, 15 mmol) in 50 mL of ethanol and allow the mixture to react at 80 °C for 12 h under nitrogen protection. After the reaction is completed, the solvent is removed under reduced pressure, and the residue is recrystallized from ethyl acetate to afford Compound **6** as a green solid (1.17 g, 85% yield). ^{1}H NMR (400 MHz, DMSO-d_6) δ 8.57 (d, J = 1.8 Hz, 2H), 8.38 (d, J = 14.1 Hz, 2H), 8.14 – 8.06 (m, 4H), 7.92 - 7.81 (m, 4H), 6.39 (d, J = 14.2 Hz, 2H), 4.36 (s, 4H), 3.03 (s, 18H), 2.77 (s, 4H), 1.94 (s, 12H), 1.80 (d, J = 7.1 Hz, 4H), 1.70 - 1.62 (m, 6H), 1.49 (s, 4H), 1.37 – 1.21 (m, 8H).

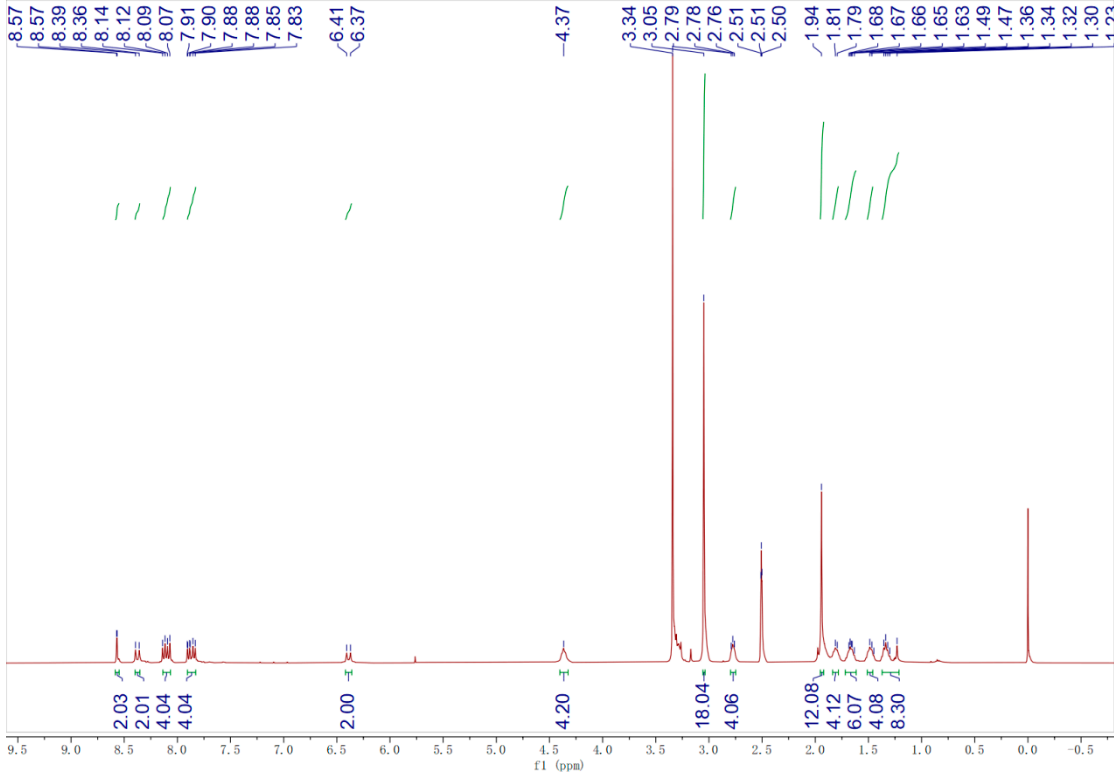

Figure S4. ^{1}H NMR spectrum (400 MHz, DMSO-*d*$_6$, 298 K) of compound **6**.

Synthesis of Compound **7**

Compound **6** (1.38 g, 1 mmol) and methylamine hydrochloride (195 mg, 3 mmol) were dissolved in 5 mL of anhydrous DMF. The mixture was reacted at 60 °C for 2 h under nitrogen protection, and then the solvent was removed under reduced pressure. The solid was dissolved by adding 20 mL of dichloromethane, followed by extraction with deionized water three times (3 × 20 mL). The organic layer was dried over anhydrous sodium sulfate, and the solvent was removed under reduced pressure to afford the crude product. Purification by column chromatography (DCM/MeOH = 10/1) gave Compound **7** as a blue solid (1.06 g, 0.78 mmol) with a yield of 77.2%. ^{1}H NMR (400 MHz, DMSO-*d*$_6$) δ 9.17 (s, 1H, NH), 8.57 (d, J = 1.8 Hz, 2H, 2ArH), 8.38 (d, J = 14.1 Hz, 2H), 8.16 - 7.95 (m, 4H), 7.92 - 7.72 (m, 4H), 6.39 (d, J = 14.3 Hz, 2H), 4.37

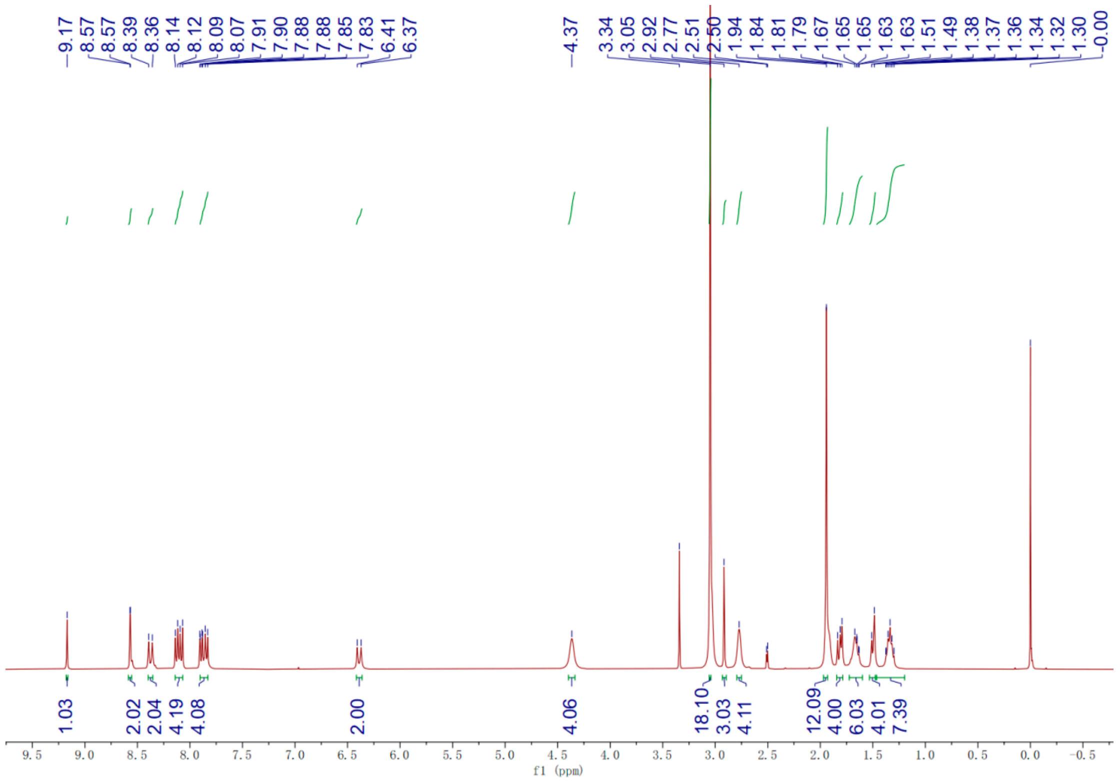

Figure S5. ^{1}H NMR spectrum (400 MHz, DMSO-d_6, 298 K) of compound **7**.

Synthesis of Compound **8**

An aqueous solution (50 mL) containing 9.7 g (16 mmol) of potassium peroxymonosulfate was added to a solution of methyl 4-aminobenzoate (1.5 g, 10 mmol) in dichloromethane (20 mL). The mixture was vigorously stirred at room temperature for 5 hours until the organic phase turned dark green. After separating the organic phase, it was washed with 0.1 N HCl (3 × 15 mL). Subsequently, the organic phase was dried over magnesium sulfate, and the solvent was removed to afford methyl 4-nitrosobenzoate as a yellow powder. Then, methyl 4-nitrosobenzoate was dissolved in DCM (15 mL) and anhydrous THF (5 mL), followed by the addition of 4-aminobenzyl alcohol (1.1 g, 10 mmol) and acetic acid (2.84 mL, 50 mmol). The reaction mixture was stirred at room temperature for 16 hours, the solvent was removed under reduced pressure, and purification by column chromatography (EA/PE = 1/10) gave compound **8** as a yellow solid (2.38 g, 88.1%).^{1}H NMR (400 MHz, DMSO-d_6) δ 8.20 - 8.13 (m,

2H), 8.01 - 7.96 (m, 2H), 7.95 - 7.88 (m, 2H), 7.57 (d, J = 8.3 Hz, 2H), 5.42 (t, J = 5.7 Hz, 1H), 4.63 (d, J = 5.6 Hz, 2H), 3.91 (s, 3H).

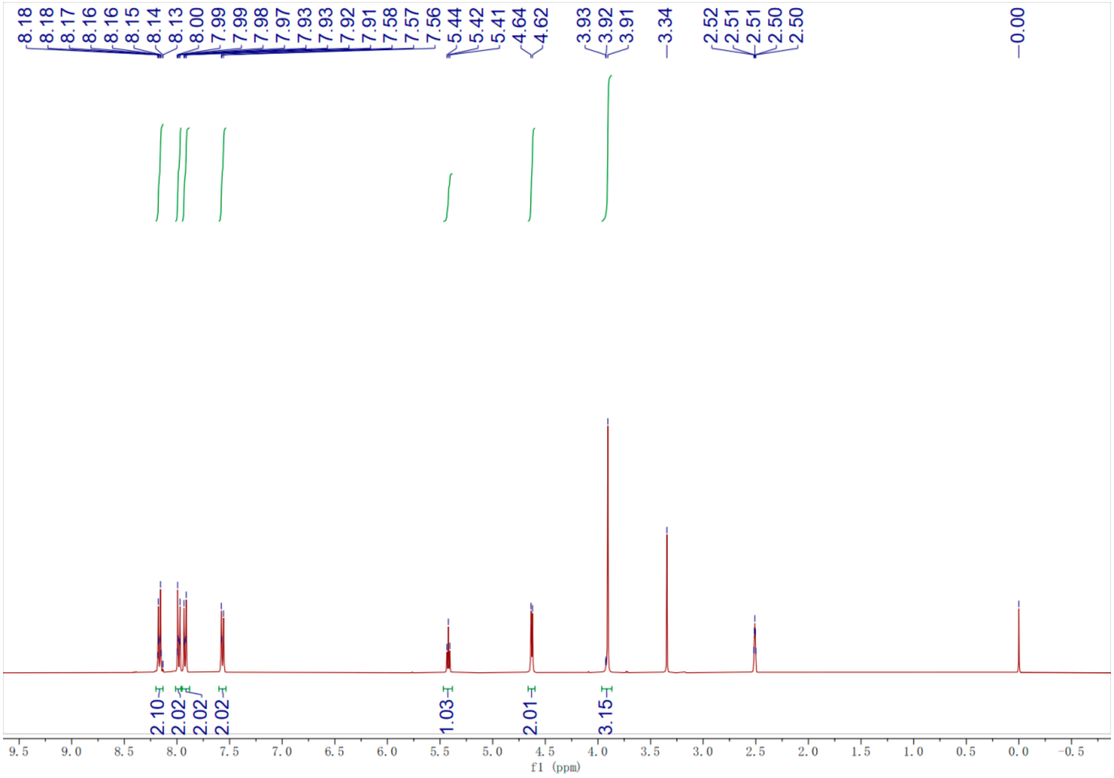

Figure S6. ^{1}H NMR spectrum (400 MHz, DMSO-d_6, 298 K) of compound **8**.

Synthesis of Compound **9**

Compound **8** (270 mg, 1 mmol) was dissolved in anhydrous THF (15 mL) under nitrogen protection. Carbon tetrabromide (703 mg, 2.12 mmol) and triphenylphosphine (556 mg, 2.12 mmol) were added, and the mixture was reacted at 60 °C for 4 hours. After the reaction was completed, the mixture was filtered, and the filtrate was purified by column chromatography (EA/n-hexane = 1/10) to afford compound **9** as an orange solid (200 mg, 60.1% yield). ^{1}H NMR (400 MHz, DMSO-d_6) δ 8.21 - 8.13 (m, 2H), 8.02 - 7.97 (m, 2H), 7.95 - 7.88 (m, 2H), 7.61 - 7.53 (m, 2H), 4.67 - 4.58 (m, 2H), 3.91 (s, 3H).

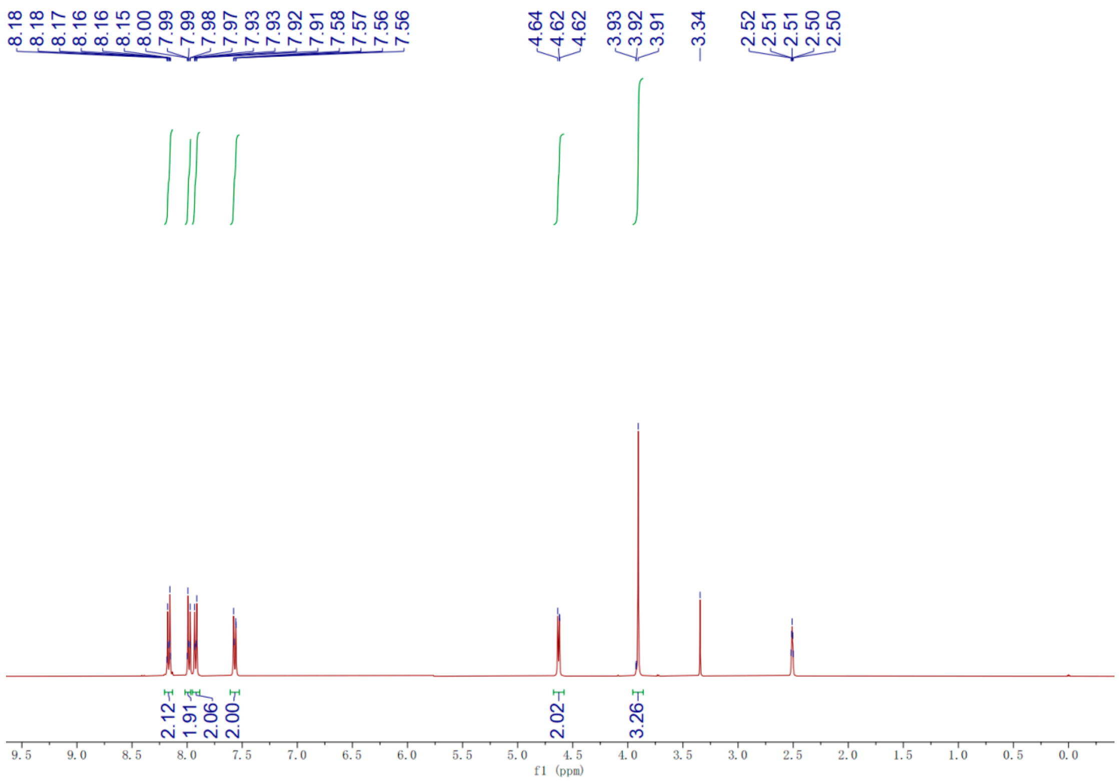

Figure S7. ^{1}H NMR spectrum (400 MHz, DMSO-d_6, 298 K) of compound **9**.

Synthesis of Compound **10**

Dissolve compound **9** (332 mg, 1 mmol) in dry DCM (10 mL), cool the solution to 0 °C, then add a mixture of lithium aluminum hydride (40 mg, 1 mmol) and 5 mL of dry THF. The reaction mixture was stirred at room temperature for 4 h, followed by dropwise addition of 2 mL of water to quench the reaction. The reaction solution was washed with saturated sodium bicarbonate solution (5 mL×3) and dried over anhydrous sodium sulfate. The solvent was removed under reduced pressure to afford the crude product. Purification by column chromatography (DCM/MeOH = 80/1) gave compound **10** as a yellow solid (280 mg, 91.8%).^{1}H NMR (400 MHz, DMSO-d_6) δ 7.93 - 7.81 (m, 4H), 7.52 (dd, J = 8.2, 6.0 Hz, 4H), 5.38 (t, J = 5.7 Hz, 1H), 4.81 (d, J = 1.1 Hz, 2H), 4.61 (d, J = 5.6 Hz, 2H).

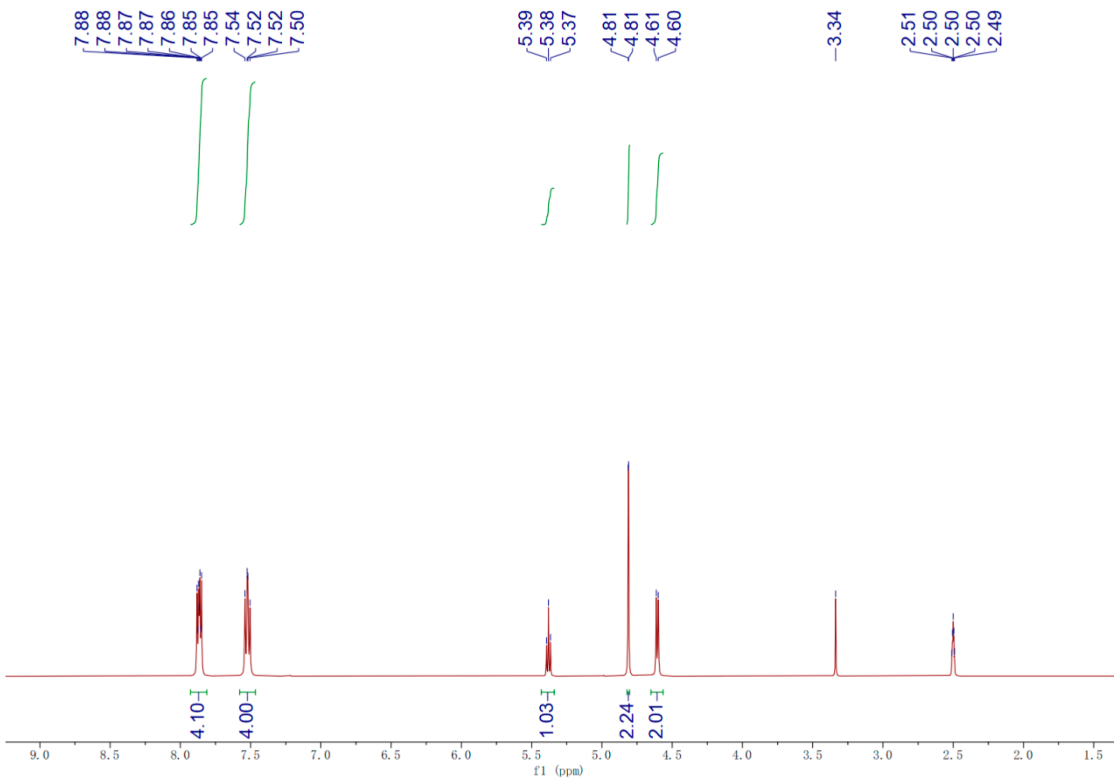

Figure S8. ^{1}H NMR spectrum (400 MHz, DMSO-*d*$_6$, 298 K) of compound **10**.

Synthesis of Compound **12**

Dissolve Compound **11** (304 mg, 1 mmol) in dry DMF (10 mL), cool the solution to 0 °C, then add NaH (120 mg, 5 mmol) in portions. Stir the mixture at room temperature for 0.5 h. Subsequently, add Compound **10** (456 mg, 1.5 mmol) and continue the reaction at room temperature for 4 h. Upon completion of the reaction, remove the solvent under reduced pressure, add 20 mL of dichloromethane, and extract with deionized water three times (3×20 mL). Collect the organic phase, dry it over anhydrous sodium sulfate, and purify by column chromatography (EA /PE = 2/1) to afford Compound **12** as a yellow solid (375 mg, 70.9%).^{1}H NMR (300 MHz, DMSO-d_6) δ 8.13 (d, J = 8.2 Hz, 1H), 7.94 – 7.86 (m, 2H), 7.83 (d, J = 8.4 Hz, 2H), 7.75 (d, J = 8.5 Hz, 1H), 7.52 (d, J = 8.5 Hz, 2H), 7.45 (m, 1H), 7.34 – 7.30 (m, 1H), 7.25 (m, 5H), 7.21 – 7.16 (m, 2H), 6.97 (d, J = 3.3 Hz, 1H), 6.48 (d, J = 3.3 Hz, 1H), 5.71 (s, 2H), 5.37 (s, 1H), 4.93 (d, J = 2.4 Hz, 2H), 4.60 (s, 2H), 4.52 (s, 2H).

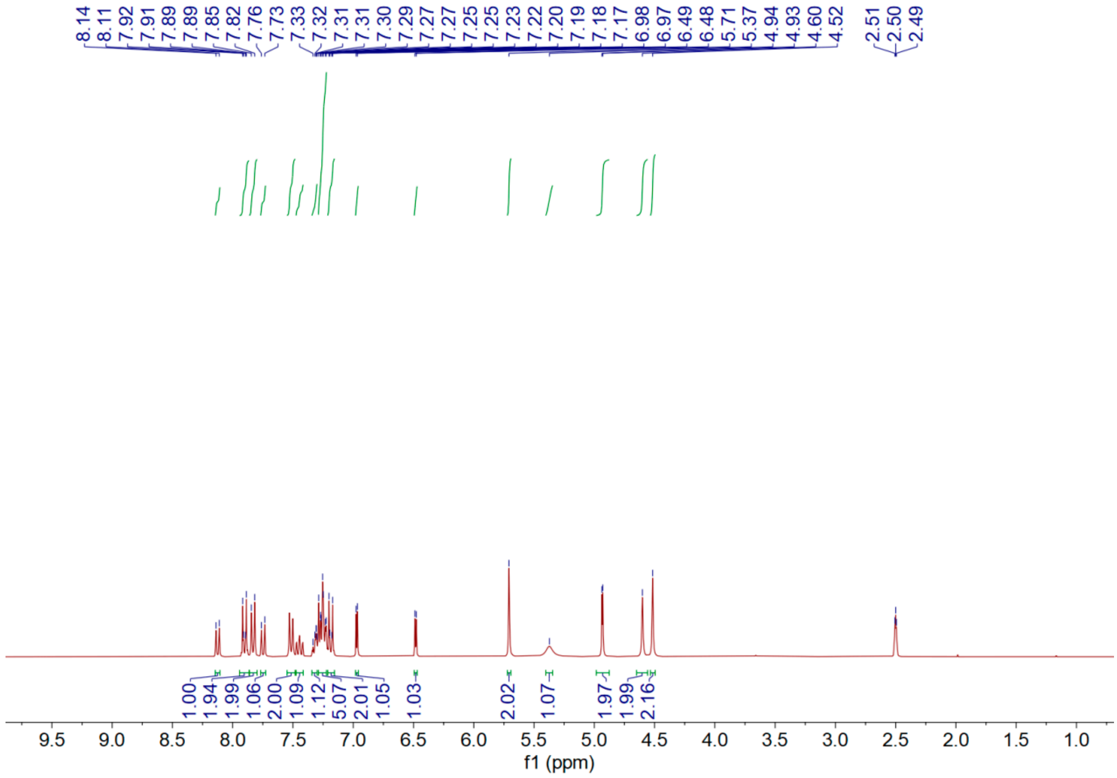

Figure S9. ^{1}H NMR spectrum (400 MHz, DMSO-d_6, 298 K) of compound **12**.

Synthesis of Compound **Cy-G**

Compound **7** (140.0 mg, 0.1 mmol) and triphosgene (12.0 mg, 0.04 mmol) were dissolved in anhydrous DCM under nitrogen protection. The mixture was then cooled to 0 °C and maintained for 10 minutes. Triethylamine (12.1 mg, 0.12 mmol) was dissolved in anhydrous DCM and added dropwise to the cooled solution. A distinct color change was observed in the solution. When the color turned completely green, the reaction was quenched with chilled saturated aqueous sodium bicarbonate solution. The solvent was subsequently removed, and the residue was redissolved in anhydrous dichloromethane. Triethylamine (30.3 mg, 0.3 mmol) dissolved in 2.0 mL of dichloromethane was added to the mixture, and the reaction was allowed to proceed for 1 hour. Compound **12** (0.16 g, 0.3 mmol) and DMAP (20 mg) were then added to the solution. The reaction progress was monitored by TLC. After completion of the reaction, the mixture was washed three times with saturated brine and extracted with dichloromethane (20 mL × 3). Purification by column chromatography (DCM/MeOH = 75/25 to 50/50) afforded the dark green solid product **13** (110 mg, 62%). ^{1}H NMR (500 MHz, DMSO-d_6) δ 8.43 (d, *J* = 1.9 Hz, 1H), 8.19 – 8.17 (m, 2H), 8.14 (d, *J* = 8.3 Hz, 2H), 8.03 – 7.98 (m, 3H), 7.97 – 7.94 (m, 3H), 7.93 (s, 1H), 7.79 (dd, *J* = 8.8, 1.9 Hz, 1H), 7.74 (s, 1H), 7.69 (d, *J* = 8.4 Hz, 3H), 7.63 (m, 1H), 7.57 (m, 2H), 7.49 – 7.38 (m, 2H), 7.32 (dd, *J* = 8.4, 6.4 Hz, 3H), 7.26 (m, 6H), 6.97 (d, *J* = 3.3 Hz, 1H), 6.49 (d, *J* = 3.3 Hz, 1H), 5.71 (s, 2H), 4.89 (s, 2H), 4.52 (s, 2H), 3.91 (s, 3H), 3.46 – 3.42 (m, 2H), 3.32 – 3.28 (m, 4H), 3.06 (s, 18H), 2.56 (dd, *J* = 10.9, 5.7 Hz, 4H), 1.90 (s, 6H), 1.75 (m, 4H), 1.70 – 1.62 (m, 4H), 1.45 (m, 4H), 1.38 – 1.28 (m, 4H), 1.20 (t, *J* = 7.3 Hz, 12H). ^{13}C NMR (101 MHz, MeOD-d_4) δ 157.4, 157.3, 157.1, 151.8, 151.7, 151.5, 151.5, 151.3, 151.3, 147.9, 147.3, 137.8, 137.7, 136.5, 136.4, 134.9, 131.1, 130.3, 129.5, 128.1, 127.5, 127.45, 126.2, 125.5, 124.2, 124.1, 62.9, 60.2, 54.5, 48.3, 48.1, 47.8, 47.6, 47.4, 47.2, 47.0, 35.1, 31.7, 29.4, 29.1, 28.9, 26.7, 25.6, 23.9, 22.4, 13.1, HRMS: (ESI, *m/z*) Calce for $C_{91}H_{104}I_2N_9O_4$ $[M]^{3+}$: 1640.6284, found 1640.6280.

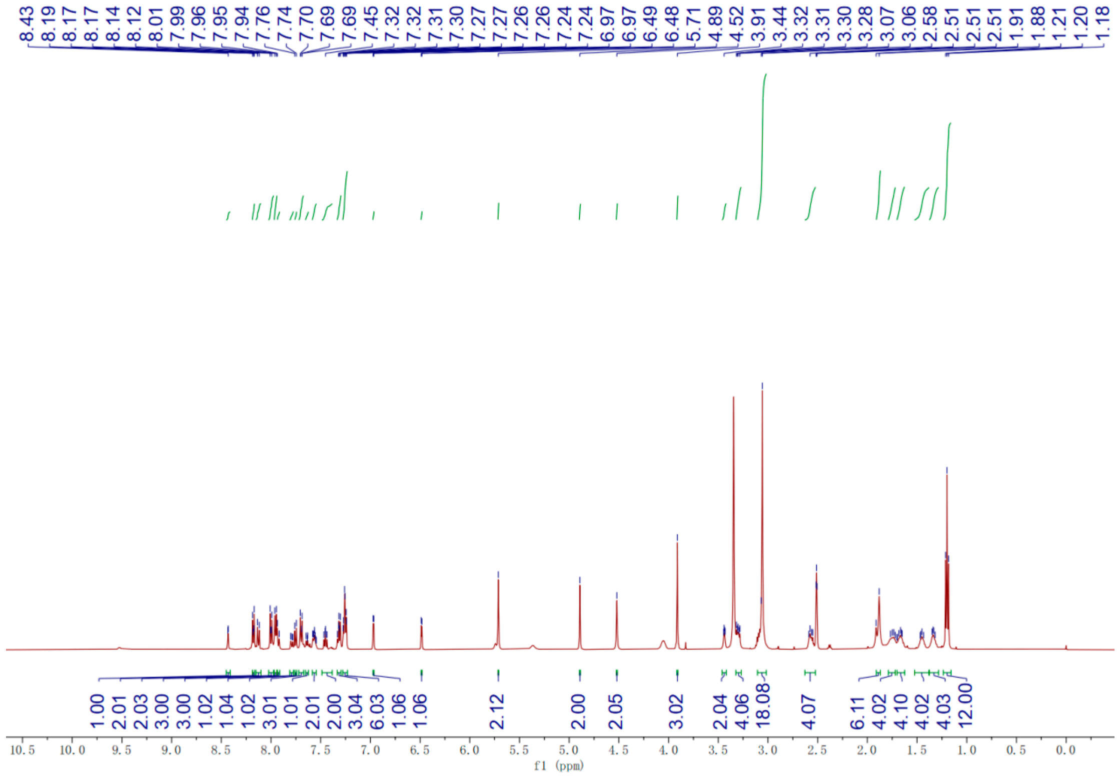

Figure S10. ^{1}H NMR spectrum (400 MHz, DMSO-d_6, 298 K) of compound **Cy-G**.

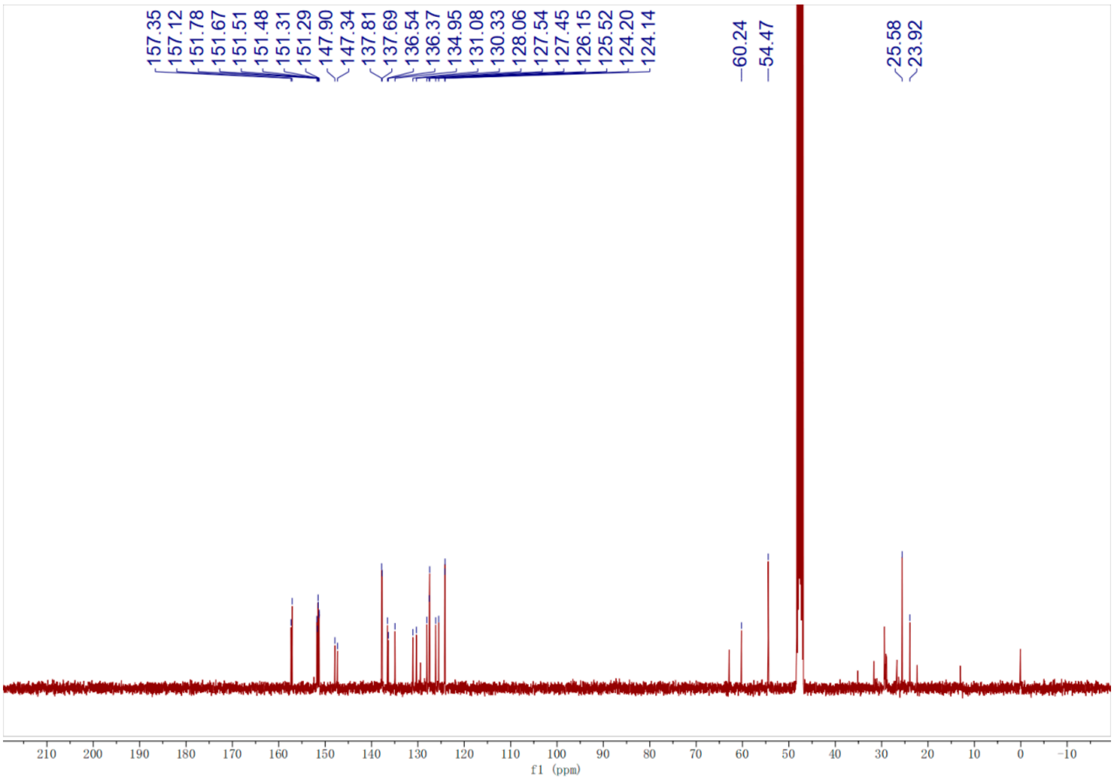

Figure S11. ^{13}C NMR spectrum (101 MHz, MeOD-d_4, 298 K) of compound **Cy-G**.

Elemental Composition Report **Page 1**

Single Mass Analysis
Tolerance = 5.0 PPM / DBE: min = -1.5, max = 50.0
Element prediction: Off
Number of isotope peaks used for i-FIT = 3

Monoisotopic Mass, Even Electron Ions
145312 formula(e) evaluated with 1 results within limits (up to 50 closest results for each mass)
Elements Used:
C: 91-91 H: 104-104 N: 9-9 O: 4-4 Cu: 0-5 Se: 0-1 Br: 0-8 I: 2-2

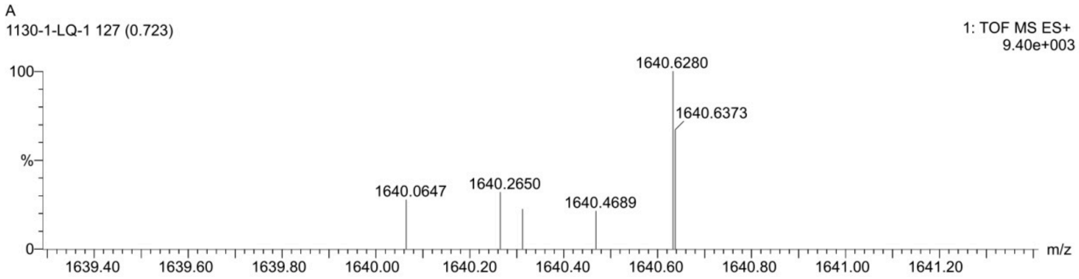

Minimum:				-1.5				
Maximum:		5.0	5.0	50.0				
Mass	Calc. Mass	mDa	PPM	DBE	i-FIT	Norm	Conf(%)	Formula
1640.6284	1640.6280	-0.4	-0.5	26.5	49.6	n/a	n/a	C91 H104 I2 N9 O4

Figure S12. HRMS of compound **Cy-G**.

4. ζ-potentials of the CyNPs

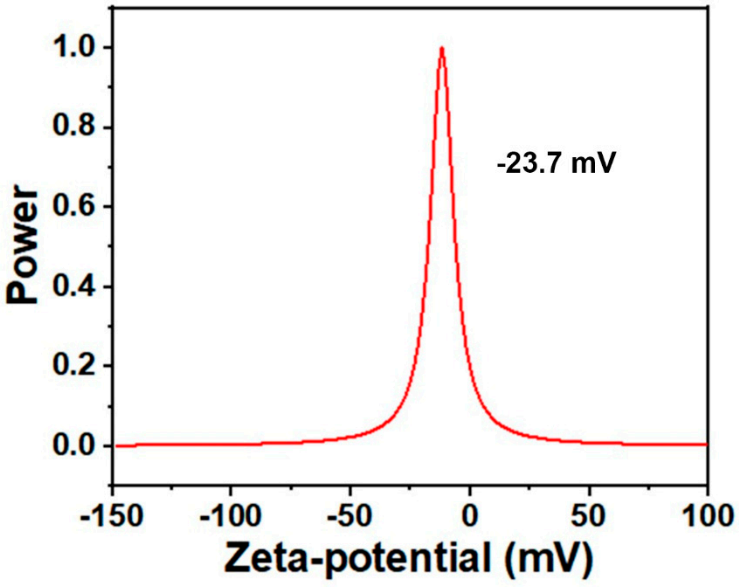

Figure S13 ζ-potentials of the CyNPs

5. ζ-potentials of the GH@CyNPs

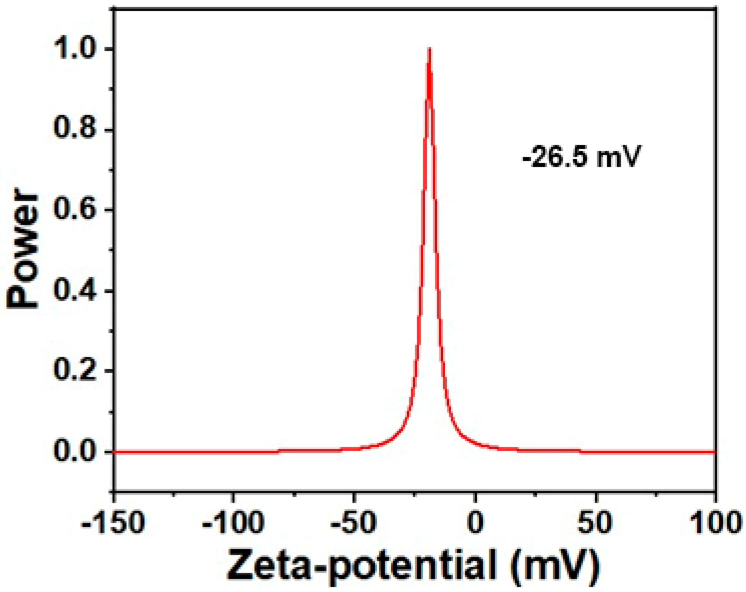

Figure S14 ζ-potentials of the GH@CyNPs

6. TEM images of CyNPs under hypoxic conditions

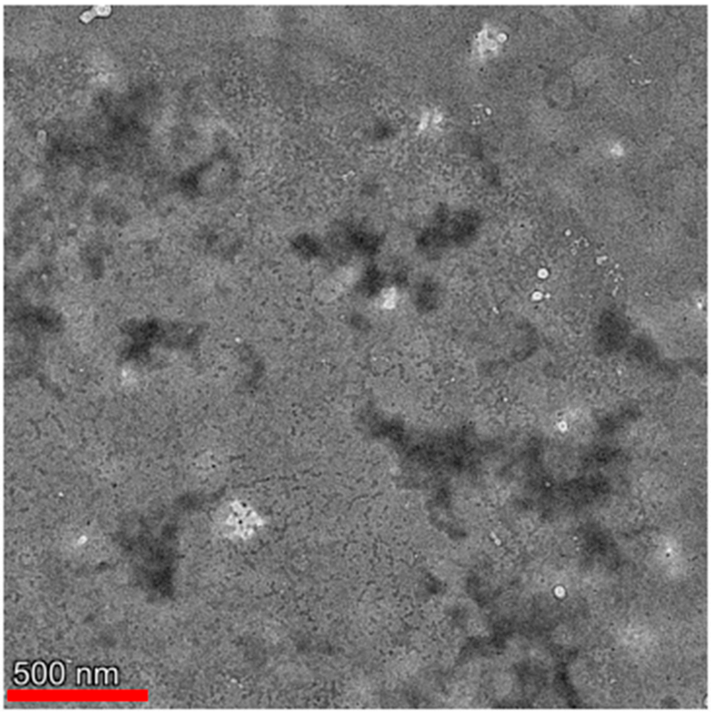

Figure S15 TEM images of CyNPs (10 μM) after incubation with rat liver microsomes and NADPH

7. Singlet oxygen generation capability of CyNPs

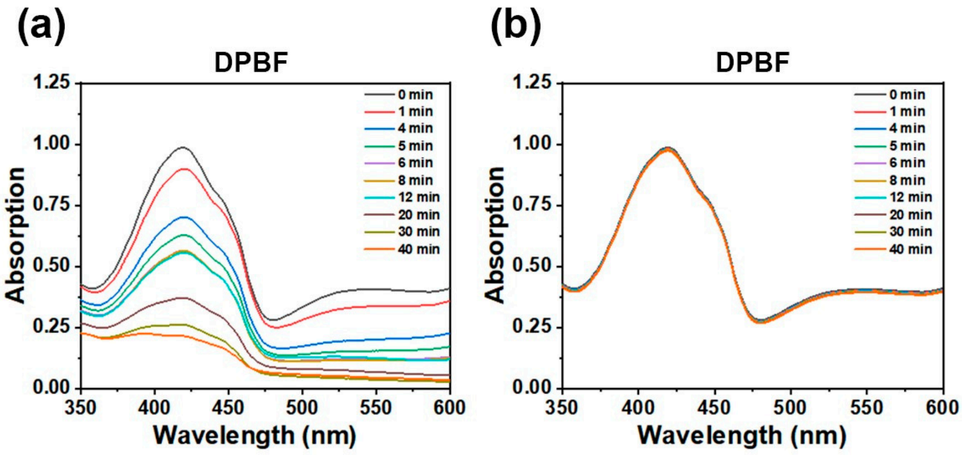

Figure S16 (a, b) $^1O_2$ generation ability of CyNPs under normoxic and hypoxic conditions upon red-light irradiation

8. Glucose-induced pH changes

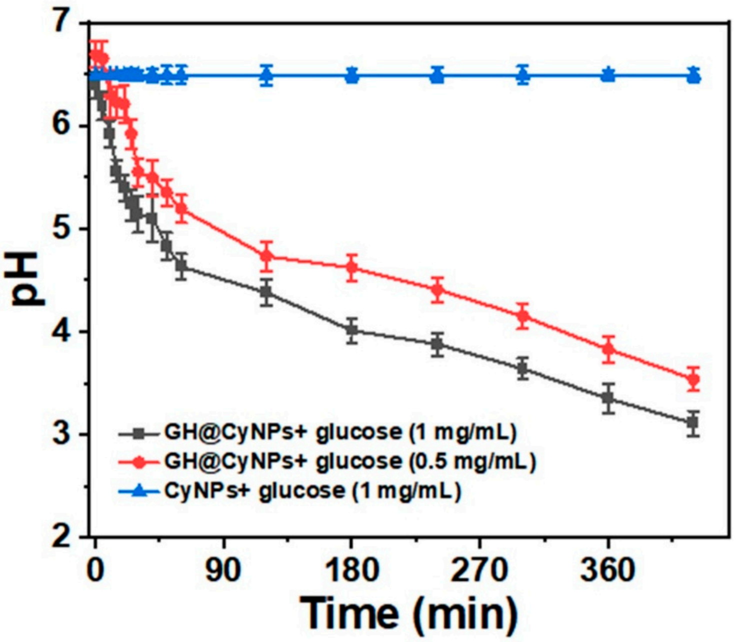

Figure *S17*. Glucose-induced pH changes.

9. Cytotoxicity

Normoxia IC50 [μM]

	HT-29	MDA-MB-231	4T1	HUVEC
GH@CyNPs	6.42	4.74	4.17	＞200
GH@CyNPs+L	5.74	4.21	3.55	＞200
CyNPs	10.14	10.79	7.09	＞200
CyNPs+L	8.88	9.07	5.76	＞200
YC-1	80.43	120.35	47.11	＞200
YC-1+L	59.13	109.79	48.51	＞200

Hypoxia IC50 [μM]

	HT-29	MDA-MB-231	4T1	HUVEC
GH@CyNPs	5.15	2.42	3.86	＞200
GH@CyNPs+L	3.63	0.89	3.62	＞200
CyNPs	42.71	18.96	5.35	＞200
CyNPs+L	8.99	3.46	4.22	＞200
YC-1	83.67	83.44	51.21	＞200
YC-1+L	211.49	46.27	41.05	＞200

Table S1. IC_{50} of different groups against HT-29 cells, MDA-MB-231 cells, 4T1 cells, and HUVEC cells.

10. Cytotoxic Mechanism

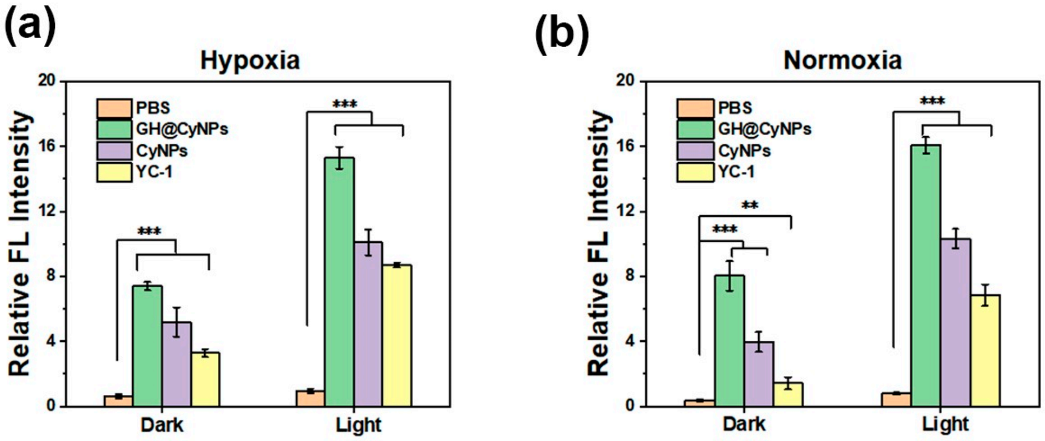

Figure S18 (a) Quantitative analysis after different administrations under hypoxia condition; (b) quantitative analysis after different administrations under normoxic condition, scale bars: 100 μm. *p < 0.05, **p < 0.01, ***p < 0.001.

11. Biosafety

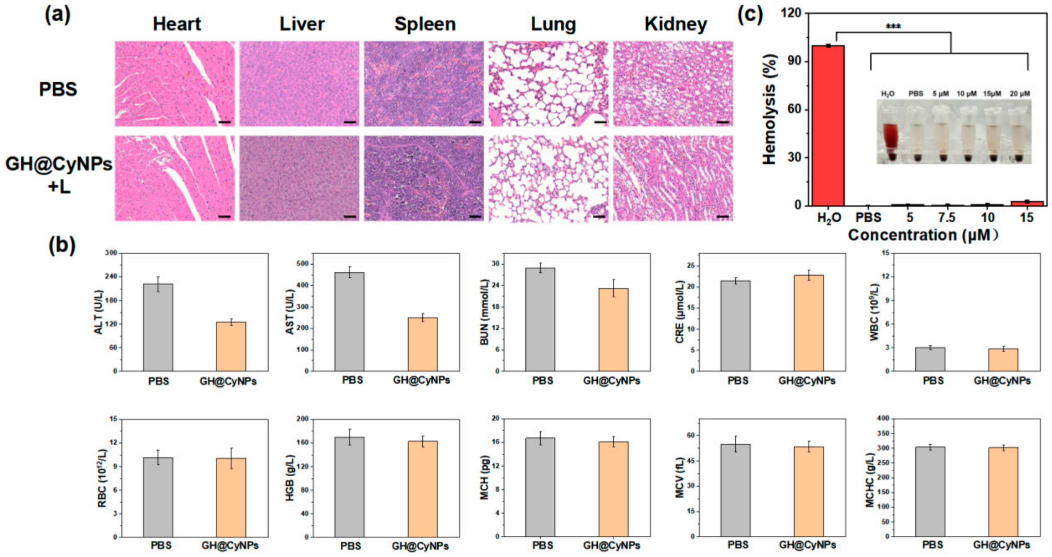

Figure S19. (a) H&E staining on major organs, scale bars: 50 μm. (b) Blood biochemical analysis of the major markers of hepatic function, renal function, and blood routine after treatment with PBS or GH@CyNPs. (c) Hemolytic test for GH@CyNPs with various concentrations. *p < 0.05, **p < 0.01, ***p < 0.001.

12. Raw data of Western blot

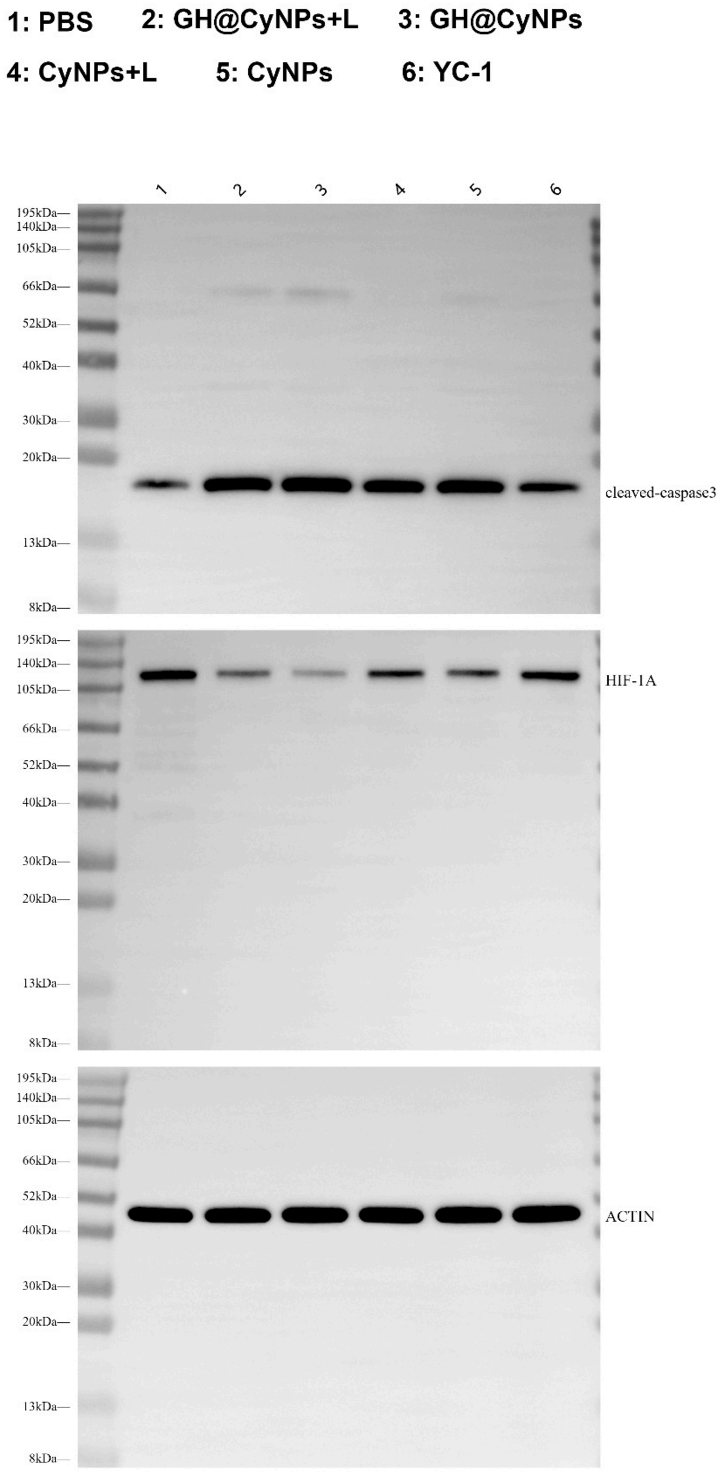

Fig. S20. Uncropped blot corresponding to Figure 4f.
